# Unconventional Pro-inflammatory CD4^+^ T Cell Response in B Cell-Deficient Mice Infected with *Trypanosoma cruzi*

**DOI:** 10.3389/fimmu.2017.01548

**Published:** 2017-11-21

**Authors:** Melisa Gorosito Serrán, Jimena Tosello Boari, Facundo Fiocca Vernengo, Cristian G. Beccaría, María C. Ramello, Daniela A. Bermejo, Amelia G. Cook, Carola G. Vinuesa, Carolina L. Montes, Eva V. Acosta Rodriguez, Adriana Gruppi

**Affiliations:** ^1^Centro de Investigaciones en Bioquímica Clínica e Inmunología (CIBICI – CONICET), Departamento de Bioquímica Clínica, Facultad de Ciencias Químicas, Universidad Nacional de Córdoba, Córdoba, Argentina; ^2^Department of Immunology and Infectious Disease, John Curtin School of Medical Research, Australian National University, Canberra, ACT, Australia

**Keywords:** *Trypanosoma cruzi*, Chagas disease, B cell, CD4^+^ T cells, inflammation

## Abstract

Chagas disease, caused by the parasite *Trypanosoma cruzi*, is endemic in Latin America but has become a global public health concern by migration of infected people. It has been reported that parasite persistence as well as the intensity of the inflammatory immune response are determinants of the clinical manifestations of the disease. Even though inflammation is indispensable for host defense, when deregulated, it can contribute to tissue injury and organ dysfunction. Here, we report the importance of B cells in conditioning T cell response in *T. cruzi* infection. Mice deficient in mature B cells (muMT mice) infected with *T. cruzi* exhibited an increase in plasma TNF concentration, TNF-producing CD4^+^ T cells, and mortality. The increase in TNF-producing CD4^+^ T cells was accompanied by a reduction in IFNγ^+^CD4^+^ T cells and a decrease of the frequency of regulatory Foxp3^+^, IL-10^+^, and IL17^+^CD4^+^ T cells populations. The CD4^+^ T cell population activated by *T. cruzi* infection, in absence of mature B cells, had a high frequency of Ly6C^+^ cells and showed a lower expression of inhibitory molecules such as CTLA-4, PD-1, and LAG3. CD4^+^ T cells from infected muMT mice presented a high frequency of CD62L^hi^CD44^−^ cells, which is commonly associated with a naïve phenotype. Through transfer experiments we demonstrated that CD4^+^ T cells from infected muMT mice were able to condition the CD4^+^ T cells response from infected wild-type mice. Interestingly, using Blimp-flox/flox-CD23icre mice we observed that in absence of plasmablast/plasma cell *T. cruzi*-infected mice exhibited a higher number of TNF-producing CD4^+^ T cells. Our results showed that the absence of B cells during *T. cruzi* infection affected the T cell response at different levels and generated a favorable scenario for unconventional activation of CD4^+^ T cell leading to an uncontrolled effector response and inflammation. The product of B cell differentiation, the plasmablast/plasma cells, could be able to regulate TNF-producing CD4^+^ T cells since their absence favor the increase of the number of TNF^+^ CD4^+^ in *T. cruzi*-infected mice.

## Introduction

Chagas’ disease is caused by the intracellular protozoan parasite *Trypanosoma cruzi*. This acute and chronic illness affects millions of people in the Americas (1) and show increasing frequency in non-endemic areas in Europe and North America due to human migration (2). During experimental *T. cruzi* infection, the innate and acquired cell-mediated immune responses, involving many cell populations such as NK cells, CD4^+^, and CD8^+^ T cells, are required for host resistance (3). These protective responses are mainly mediated by cytokines such as TNF and IFNγ, which activate macrophages to destroy ingested parasites and to release pro-inflammatory cytokines (4–8). Impaired production of pro-inflammatory cytokines as observed in mice lacking functional myeloid differentiation factor 88 lead to decreased host resistance to *T. cruzi* acute infection (9). However, uncontrolled accumulation of pro-inflammatory cells may induce tissue damage of the infected host (10–14). Models of experimental *T. cruzi* infection using genetically engineered mice such as IL17RA-deficient mice (15) or WSX-1 (IL-27R)-deficient mice (16) showed that a deregulated pro-inflammatory cytokine production results in increased susceptibility to *T. cruzi* infection. Then, the inflammatory response must be properly balanced; it has to be strong enough to control the pathogen but tightly controlled to minimize immune-mediated pathology (17, 18).

Different players have been implicated in the immune regulation during *T. cruzi* infection, such as anti-inflammatory cytokines, like IL-10 and TGF-β, Foxp3^+^ regulatory T cells (Treg cells), and endogenous glucocorticoids (19, 20). Indeed, deficient signaling of IL-10 correlated with increased mortality in experimental *T. cruzi* infection due to overwhelming inflammatory responses mediated by TNF and IFNγ (21, 22). Depletion of Treg cells in *T. cruzi*-infected mice leads to reduced cardiac parasitosis and inflammation, accompanied by an augmented Th1 response early in the course of infection followed by a downregulation of this response and increased Th17 response late in infection (23).

During the past years, several evidences demonstrated that B cells can influence T cell responses as antigen-presenting cells or regulatory cells (24, 25). In this regard, during *T. cruzi* infection, B cells provide parasite-specific Abs which are key for trypomastigotes control (26) and also produce cytokines that can influence cellular immunity (27, 28). Besides these reports, the complete picture of the B cell function in *T. cruzi* infection has not been deeply characterized. In this study, we analyzed the characteristics of the CD4^+^ T cell response generated in absence of B cells during experimental Chagas disease. Our results demonstrated that the T cell response induced by *T cruzi* in the absence of mature B cells, and consequently in their product of differentiation plasmablast/plasma cells, exhibit an unconventional pro-inflammatory profile, highlighting a critical role of B cells during this parasite infection.

## Materials and Methods

### Ethic Statement

All animal experiments were approved by and conducted in accordance with guidelines of the committee for Animal Care and Use of the Facultad de Ciencias Quimicas, Universidad Nacional de Cordoba (Approval Number HCD 1525/14) in strict accordance with the recommendation of the Guide to the Care and Use of Experimental Animals published by the Canadian Council on Animal Care (OLAW Assurance number A5802-01).

### Mice

C57BL/6 CD45.1 mice (B6.SJL-*Ptprca Pepcb*/Boy) and muMT mice (B6.129S2-Ighmtm1Cgn/J) were initially obtained from The Jackson Laboratories (USA) and C57BL/6 (CD45.2) mice, indicated as wild-type (WT) along the manuscript, were obtained from Facultad de Veterinaria, Universidad Nacional de La Plata (La Plata, Argentina). These animals were housed and bred in the Animal Facility of the CIBICI-CONICET, Facultad de Ciencias Quimicas, Universidad Nacional de Cordoba.

Blimp-flox/flox-CD23icre mice were obtained from crossing of females Blimp-flox/flox-CD23icre-negative and males Blimp-flox/flox-CD23icre-positive. Blimp-flox/flox-CD23icre mice are plasmablast/plasma cell-deficient mice. iCre-negative littermates were used as controls (plasmablast/plasma cell sufficient). These animals were housed and bred in the Animal Facility of the John Curtin School of Medical Research, Australian National University, Canberra, ACT, Australia.

### Parasites and Experimental Infection

*Trypanosoma cruzi* parasites (Y-Br strain) were cultured in NIH3T3 mouse fibroblasts and were collected as described (29). Mice 7–9 weeks of age were infected by intraperitoneal injection of 1 × 10^4^ trypomastigotes diluted in a solution of 1% glucose in PBS (28). Uninfected normal littermates were injected with 1% glucose in PBS and processed in parallel. Parasitemia was monitored by counting the number of viable trypomastigotes in blood after lysis with a 0.87% ammonium chloride buffer. Tissues were collected at different days post infection (Dpi) for parasite DNA quantification and T cell response analysis. Livers were collected for histological studies. Survival and weight of each mouse was followed every day and every 3 days, respectively.

In all figures, infected WT mice are indicated with empty circles or in black and infected muMT mice are indicated in blue.

### Body Weight Determination

The body weight of mice infected with *T. cruzi* was scored with a laboratory scale Scout Pro (OHAUS). Mice were individually identified and weighted just before and after infection. That initial weight was considered 100%. Every 3 days, the weight of each mouse was registered and related to its initial one, obtaining the percentage of the day of the determination.

### Quantification of Parasite DNA in Tissues

Genomic DNA was purified from 50 µg of tissue (heart, liver, and spleen) using TRIzol Reagent (Life Technologies) following the manufacturer’s instructions. Satellite DNA from *T. cruzi* (GenBank AY520036) was quantified by real-time PCR using specific Custom Taqman Gene Expression Assay (Applied Biosystem) using the primer and probe sequences described by Piron et al. (30). A sample was considered positive for *T. cruzi* when the threshold cycle (CT) for the *T. cruzi* target was <45. Abundance of satellite DNA from *T. cruzi* was normalized to GAPDH abundance (Taqman Rodent GAPDH Control Reagent, cat. code 4308313, Applied Biosystem) and expressed as arbitrary units.

### Cell Preparation

Blood, spleens, inguinal lymph nodes, and livers were obtained, and tissues were homogenized through a tissue strainer. Erythrocytes in blood and spleen and liver cell suspensions were lysed for 5 min in Tris–ammonium chloride buffer. Liver-infiltrating cells were obtained after 20 min centrifugation (600 *g*) in a 30 and 70% bilayer Percoll (Sigma) gradient (15). Viable cell numbers were determined by trypan blue exclusion using a Neubauer counting chamber.

### Cytokine Quantification

Cytokine concentrations in plasma were assessed by ELISA using paired Abs for mouse IL1-b, IL-12p70, IL-6, IFNγ (BD Biosciences, USA), and TNF and IL10 (Biolegend, USA) according to the manufacturer’s instructions.

### Flow Cytometry

Cell suspensions were washed in ice-cold FACS buffer (PBS-2% FBS) and incubated with fluorochrome labeled-Abs for 20 min at 4°C. Different combinations of the following anti-mouse Abs (BD Biosciences, eBioscience, Biolegend) were used: FITC-labeled anti-CD11b (M1/70), anti-CD3 (145-2C11), anti-CD44 (IM7); PE-labeled anti-CD4 (GK1.5), anti-CXCR3 (CXCR3-173), anti-2B4 (eBio244F4); APC or eFluor660-labeled anti-Ly6C (HK1.4), anti-CD39 (24DMS1); PerCP-eFluor710 or PerCpCy5.5-labeled anti-Ly6C (HK1.4), anti-CD62L (MEL-14), anti-TIGIT (GIGD7), anti-CD39 (24DMS1), anti-LAG3 (C9B7W), anti-CD45.2 (104); PECy7-labeled anti-PD1 (RMP1-30), APC-eFluor 780 or APCCy7-labeled anti-CD4 (GK1.5), anti-CD45.1 (A20). Transcription factors and CTLA4 were detected after cell fixation and permeabilization with Foxp3 Staining Buffer Set according to the manufacturers’ protocol (eBioscience) using the following antibodies: PE-labeled anti-Tbet (eBio4B10), anti-CTLA4 (UC10-4B9), and anti-Foxp3 (FJK-16s); APC-labeled anti-Foxp3 (FJK-16s); PerCPCy5.5-labeled anti-Foxp3 (FJK-16s). For intracellular cytokine staining, cells were cultured for 5 h with 50 ng/ml PMA (phorbol 12-myristate 13-acetate) (Sigma), 1,000 ng/ml ionomycin (Sigma) and Brefeldin A (eBioscience) or Monensin (eBioscience). Cells were fixed and permeabilized with BD Cytofix/Cytoperm and Perm/Wash (BD Biosciences) according the manufacturer’s instructions. Cells were incubated with surface-staining antibodies and PE-labeled anti-TNF (MP6-XT22), anti-IFNγ (XMG1.2), anti-IL10 (JES5-16E3), anti-IL17 (eBio17B7), and PerCP-labeled anti-TNF (MP6-XT22). Cells were acquired on FACSCanto II (BD Bioscience) and were analyzed using the FlowJo software (TreeStar).

Along the manuscript, the results were expressed as percentage because the initial cellularity of the lymphoid tissues of muMT mice is significantly different than that of WT mice. The size of the spleens and lymph nodes of muMT mice is significantly lower than that of the WT mice. After infection, spleen size of muMT mice increased significantly but did not reach the size of the spleens of infected WT mice. The percentage shows the actual changes in the frequency of cells and indicates how much a population is over- or sub-expressed respect to another (Figure S1 in Supplementary Material).

### Liver Histology

Livers obtained from WT and muMT mice at different days post *T. cruzi* infection were fixed in formaldehyde 4% and embedded in paraffin. Five-millimeter thick sections were examined by light microscopy after hematoxylin/eosin (HE) staining. Double-blind evaluation of liver damage focused in three main aspects: presence and type of inflammatory infiltrate, presence and extension of necrotic areas and hyaline degeneration, and presence of cellular alterations such as vacuolization, swelling, and nuclear alterations. Photographs were taken using a Nikon Eclipse TE 2000 U equipped with a digital video camera.

### Transaminase Activity and Glucose

Plasma alanine aminotransferase (ALT) activity and plasma glucose concentrations were measured using commercial kits (Wiener Lab) following the manufacturer’s instruction.

### T Cell Adoptive Transfer

C57BL/6 CD45.1 and C57BL/6 CD45.2 (indicated along the manuscript as WT) and muMT (CD45.2) mice were infected with 1 × 10^4^ trypomastigotes diluted in a solution of 1% glucose in PBS (see schedule in Figure 8). At 9 Dpi, WT- and muMT-infected mice were sacrificed and their spleens were obtained and homogenized through a tissue strainer. Erythrocytes in spleen cell suspensions were lysed for 5 min in Tris–ammonium chloride buffer. Splenocytes were washed with MACS buffer (0.5% fetal bovine serum, 50 mM EDTA in PBS) and CD4^+^ T cells were purified using CD4 (L3T4) MicroBeads for positive selection according to the manufacturers’ guidelines (Miltenyi Biotec). CD4^+^ T viable cell numbers were determined by trypan blue exclusion using a Neubauer counting chamber. Purified CD4^+^ T cells (2 × 10^6^ from WT- or muMT-infected mice) resuspended in PBS were injected i.v. in the retro orbital sinus of C57BL/6 CD45.1 mice. C57BL/6 CD45.1 mice that received CD4^+^ T cells from WT (CD45.2) were named WT + WT and those who received CD4^+^ T cells from infected muMT mice were named WT + muMT. In addition, infected WT and muMT mice were injected i.v. with PBS as controls. At 15 Dpi, WT (*n* = 8), muMT (*n* = 4) (control groups), WT + WT (*n* = 4), and WT + muMT (*n* = 5) mice were sacrificed and lymph nodes and spleens were obtained for evaluation. Gated CD45.1 CD4^+^ T cells were analyzed for TNF and Ly6C expression.

### Statistics

Statistical significance through comparison of mean values was assessed by a two-tailed Student’s *t*-test, one-way or two-way ANOVA followed by Bonferroni’s posttest using GraphPad software.

## Results

### muMT Mice Controlled Parasite Replication but Presented High Liver Damage during *T. cruzi* Infection

**Figure 1:**
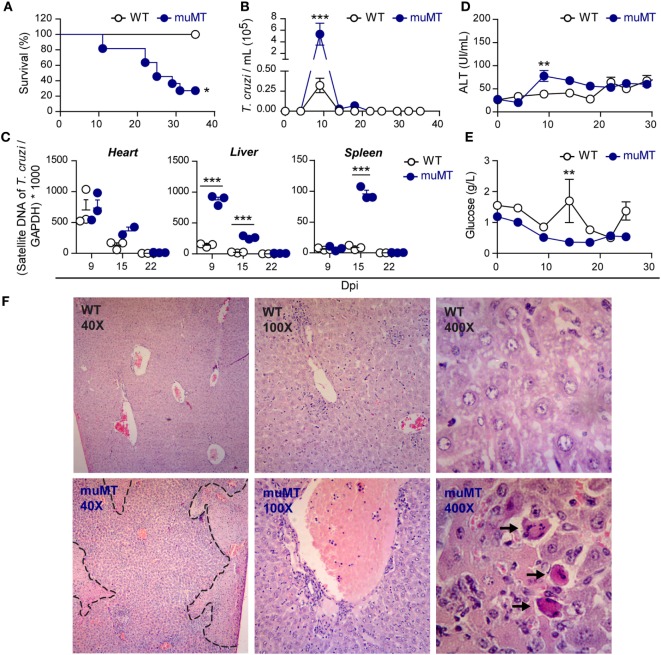
*Trypanosoma cruzi*-infected muMT mice controlled parasite replication but presented increased liver damage. Wild-type (WT) (*n* = 9, empty circles) and muMT (*n* = 13, blue circles) mice were infected with 10,000 trypomastigotes of *T. cruzi* Y strain. **(A)** Percentage of survival vs. days post infection (Dpi) (*p < 0.05 Gehan–Breslow–Wilcoxon test). **(B)** Number of trypomastigotes in blood samples at different Dpi (***p < 0.001, two-way ANOVA, Bonferroni post-test). **(C)** Parasite burden determined by RT-PCR of satellite DNA of *T. cruzi* relative to GADPH (three replicates each Dpi) (***p < 0.001, one-way ANOVA, Bonferroni post-test) in heart, liver, and spleen. **(D)** Alanine aminotransferase activity in plasma samples obtained at different Dpi (**p < 0.01, two-way ANOVA, Bonferroni post-test). **(E)** Glucose concentration in plasma samples at different Dpi (**p < 0.01, two-way ANOVA, Bonferroni post-test). Zero Dpi refers to uninfected mice. **(F)** Representative photographs of liver sections obtained at 9 Dpi and stained with hematoxilin–eosin. WT: upper panels; muMT: lower panels. The dashed line surrounds a necrotic area. Arrows indicate Councilman Bodies. Experiment representative of 3.

To deeply evaluate the disease progression and T cell response during *T. cruzi* infection in mice deficient in mature B cells, WT and muMT mice were infected with Y strain trypomastigotes of *T. cruzi*. Parasitemia, tissue parasitism, and damage in vital organs were evaluated. As previously reported (28, 31), muMT mice were more susceptible than WT mice to *T. cruzi* infection, as most of them succumbed after 20 Dpi (Figure 1A). We observed that muMT mice presented higher levels of parasitemia at 9 Dpi (which corresponds to the peak) than infected WT mice, but later controlled parasite levels to the same extent and with the same kinetics as WT mice did (Figure 1B). When we studied parasite load in target organs of the infection, we determined that heart parasite load was similar in muMT and WT at all times studied; however, muMT mice displayed a higher level of *T. cruzi* DNA in liver (9 and 15 Dpi) and spleen (15 Dpi) in comparison to infected WT mice (Figure 1C). Later, at 22 Dpi, the parasitism in the organs of muMT mice decreased, reaching similar values to infected WT mice (Figure 1C). These data indicate that although muMT mice succumbed to the infection, they were able to control parasite replication, suggesting that uncontrolled parasitism is not the cause of death.

To evaluate organ damage, we focused on a target organ as the liver and determined the plasma activity of the liver transaminase ALT (Figure 1D). ALT activity, in infected WT mice, was similar along *T. cruzi* infection. In contrast, muMT mice showed a significantly higher activity of ALT at 9 Dpi (Figure 1D) indicating that, in absence of B cells, liver damage occurs at an earlier time pi. In the same direction, muMT-infected mice presented an altered plasma glucose kinetic. While WT-infected mice had an increase in plasma glucose at 15 Dpi, infected muMT mice presented decreased values along the acute phase and no increase at 15 Dpi (Figure 1E). The data from uninfected mice were indicated as 0 Dpi.

By histological analysis, we observed that the liver from acutely infected muMT mice showed more severe lesions in comparison to those from infected WT mice. Livers from infected WT mice obtained at 9 Dpi presented mild multifocal and perivascular leukocyte infiltrates and Kupffer cell hyperplasia (Figure 1F, upper photographs). In the absence of mature B cells, livers of infected mice presented necrosis, with vascular thrombosis and inflammatory infiltrates in venous vessels, which were dilated and full of blood (Figure 1F, lower photographs). In addition, individual lesions of hepatocytes were observed. Councilman bodies (indicated with arrows) could be observed only in infected muMT mice, suggesting that hepatocytes are undergoing apoptosis (32). At 20 Dpi, liver from infected muMT mice presented lesser necrosis and individual-cell degenerative changes but more overall damage than WT controls did (data not shown). From the analysis of results in Figure 1, we concluded that the liver of infected muMT mice exhibited a greater damage at 9 Dpi likely due to the increased parasite load. However, considering that the damage continued along infection, we hypothesized that liver immunopathology could be a consequence of the effector immune response induced by the infection.

### *T. cruzi*-Infected muMT Mice Presented High Levels of TNF and Body Weight Loss

**Figure 2:**
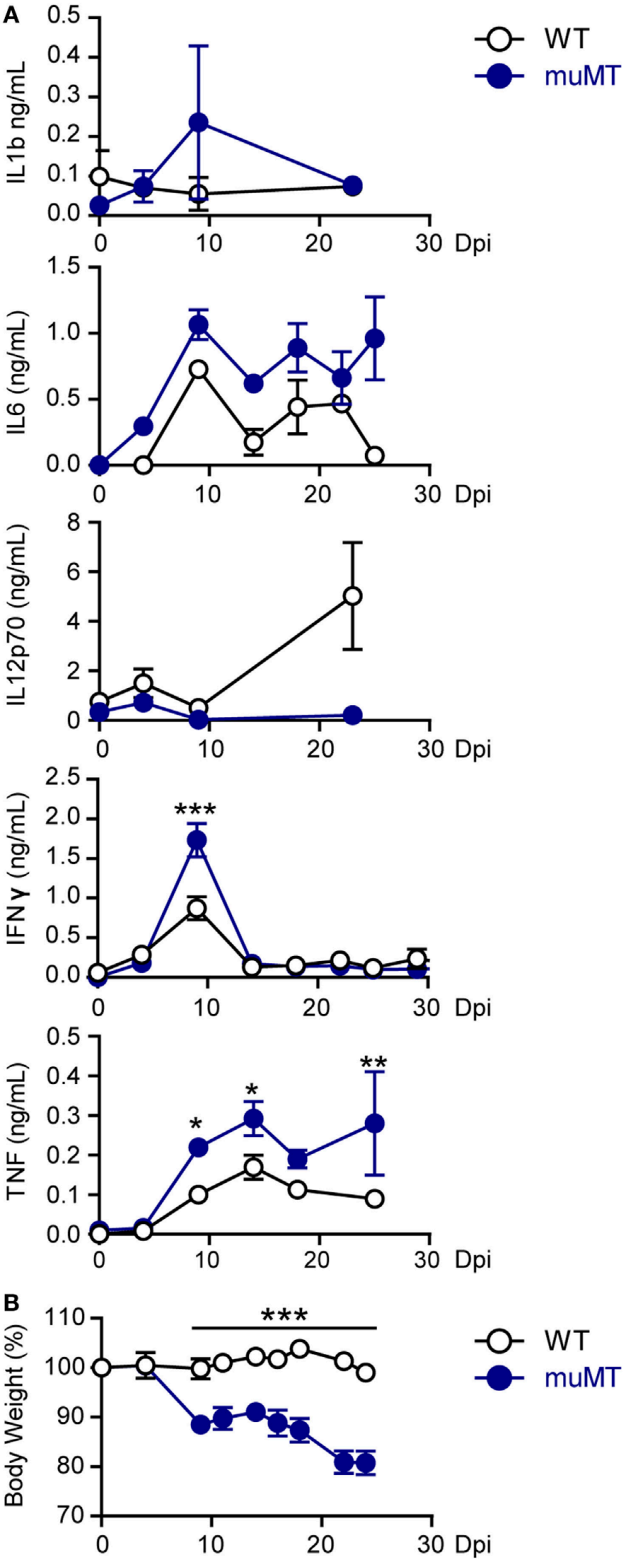
*Trypanosoma cruzi*-infected muMT mice exhibited high levels of TNF in plasma and body weight loss. Wild-type (WT) (*n* = 9, empty circles) and muMT (*n* = 13, blue circles) mice were infected with 10,000 trypomastigotes of *T. cruzi* Y strain. **(A)** Concentrations of IL-1b, IL-6, IL-12p70, INFγ, and TNF in plasma at different days post infection (Dpi). **(B)** Body weight of infected WT or muMT mice at different Dpi (100% was considered at 0 Dpi). Data are shown as mean ± SD (*p < 0.05, **p < 0.01, ***p < 0.001, two-way ANOVA, Bonferroni post-test). Experiment representative of 4.

Taking into account that organ damage can be a consequence of deregulated inflammatory responses (10, 11, 33), we next evaluated the levels of pro- and anti-inflammatory cytokines such as IL-1β, IL-6, IL-12, TNF, IFNγ, and IL-10 in plasma of *T. cruzi*-infected WT and muMT mice. Along the infection, in both experimental groups, there were no significant differences in the concentrations of IL-1β, IL-12, and IL-6 (Figure 2A) and the levels of IL-10 were undetectable (data not shown). At 9 Dpi, the plasma of infected muMT mice presented significantly higher concentrations of IFNγ and TNF (Figure 2A) than the plasma of WT infected mice. By 15 Dpi, the plasma levels of IFNγ decreased in all infected mice (Figure 2A). Of note, infected muMT mice presented significantly higher concentrations of TNF in plasma than infected WT mice from 9 Dpi until their death.

The cytokine profile observed in infected muMT mice was associated with hepatocyte damage (34, 35) and this is consistent with the liver injury observed in infected muMT mice (Figure 1D) and was accompanied with body weight loss (Figure 2B). Strengthening the idea that the great inflammatory response is associated with the death of the infected muMT mice, we noticed a link between high plasma concentration of TNF and occurrence of death (Figure S2 in Supplementary Material).

### CD4^+^ T Cells Were the Major Producers of TNF in *T. cruzi*-Infected muMT Mice

**Figure 3:**
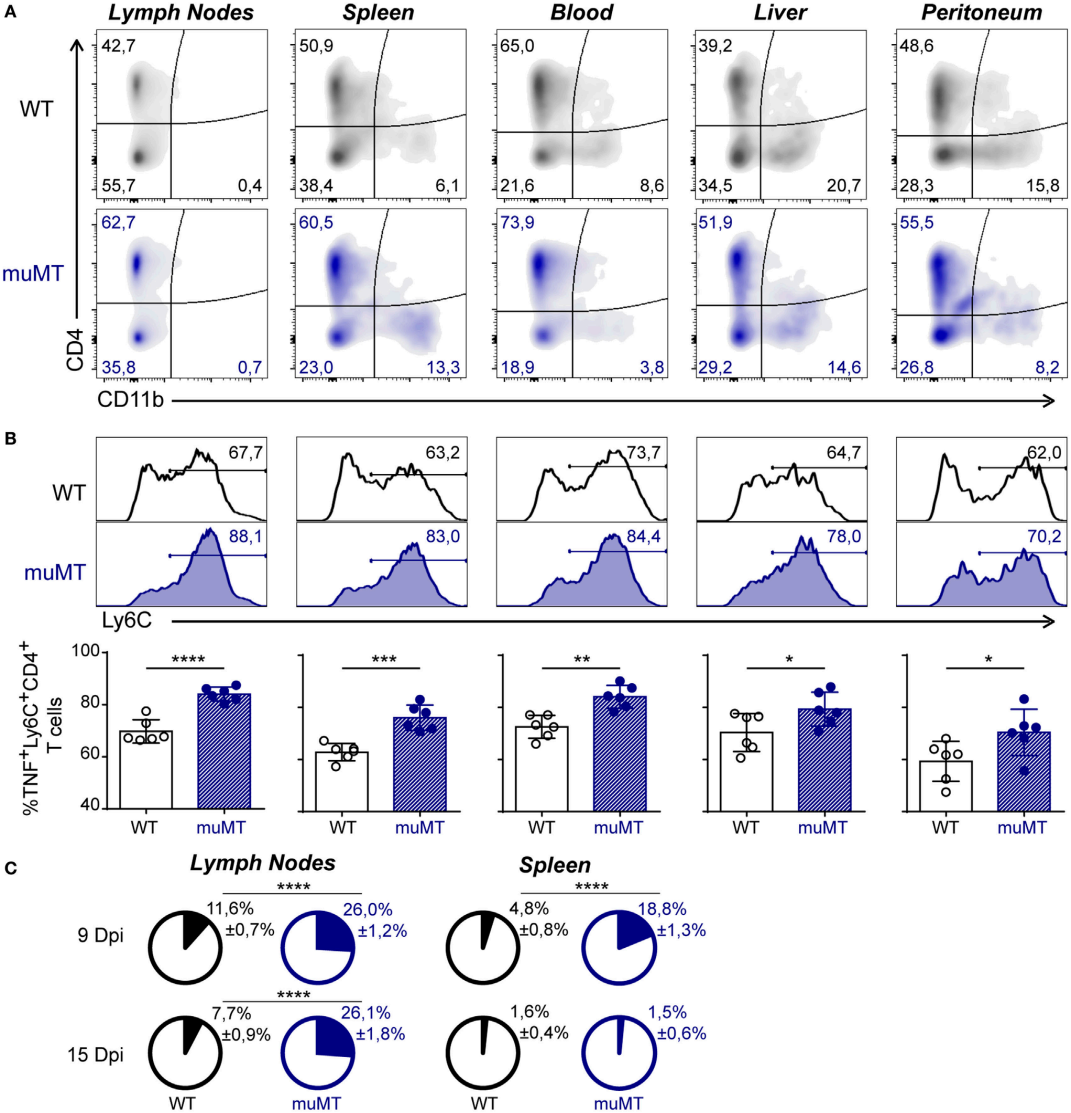
Ly6C^+^CD4^+^ T cells were the main TNF-producing cells in *Trypanosoma cruzi*-infected muMT mice. Wild-type (WT) (*n* = 6, in black) and muMT (*n* = 6, in blue) mice were infected with 10,000 trypomastigotes of *T. cruzi* Y strain. **(A)** Representative dot plot showing the percentage of CD4^+^ vs. CD11b^+^ cells, gated on TNF^+^ cells in lymph nodes, spleen, blood, liver, and peritoneum obtained from infected WT or muMT mice at 15 days post infection (Dpi). **(B)** Representative histograms of Ly6C^+^ cells on TNF-producing CD4^+^ T cells (numbers indicate the percentages of Ly6C^+^ cells) and statistical analysis of the mean ± SD of the percentages of TNF^+^Ly6C^+^CD4^+^ T cells, relative to CD4^+^ T cells, in the mentioned tissues. **(C)** Pie chart showing the mean proportion ± SD of TNF^+^Ly6C^+^CD4^+^ cells (colored slice) respect to the total cell number of lymph nodes and spleen obtained at 9 and 15 Dpi (*p < 0.05, **p < 0.01, ***p < 0.001, ****p < 0.0001; two-tailed *t*-test). Experiment representative of 3.

As the aforementioned result indicates that infected muMT mice present an imbalance in systemic inflammation, and considering that it was reported that a high production of systemic TNF significantly correlates with mortality in *T. cruzi*-infected mice (22), we first evaluated which cells can produce this cytokine. We performed flow cytometry on gated TNF-producing leukocytes (Figure S3 in Supplementary Material shows gated strategy) obtained from lymph nodes, spleen, blood, liver, and peritoneum. We observed that, in 15 Dpi-infected muMT mice, more than 50% of the TNF-producing cells were CD4^+^ cells and the second major TNF^+^ population was CD4^−^CD11b^−^. A minor subset of the TNF-producing cells was CD11b^+^ cells (Figure 3A). CD4^+^ cells were CD3^+^ T cells while CD4^−^CD11b^−^ cells were mainly CD8^+^CD3^+^ T cells (data not shown).

Remarkably, among the TNF-producing CD4^+^ T cells, 15 Dpi-infected muMT mice presented an increased percentage of Ly6C^+^ cells (Figure 3B), which have been associated with an exacerbated Th1 profile (36, 37). TNF^+^Ly6C^+^CD4^+^ T cells were significantly represented in the lymph nodes and spleen of infected muMT mice at 9 Dpi and in lymph nodes at 15 Dpi (Figure 3C).

### B Cell Deficiency in *T. cruzi* Infection Reduced the Frequency of Th1, Th17, and IL-10^+^CD4^+^ T Cells

Considering that infected muMT mice presented an increase in TNF in plasma, and that the main producers of this cytokine were CD4^+^ T cells expressing Ly6C, we evaluated whether the CD4^+^ T cell response was polarized toward a Th1 response. To asses this, we evaluated in CD4^+^ T cells of infected mice the production of IFNγ and TNF, and the expression of T-bet and CXCR3.

**Figure 4:**
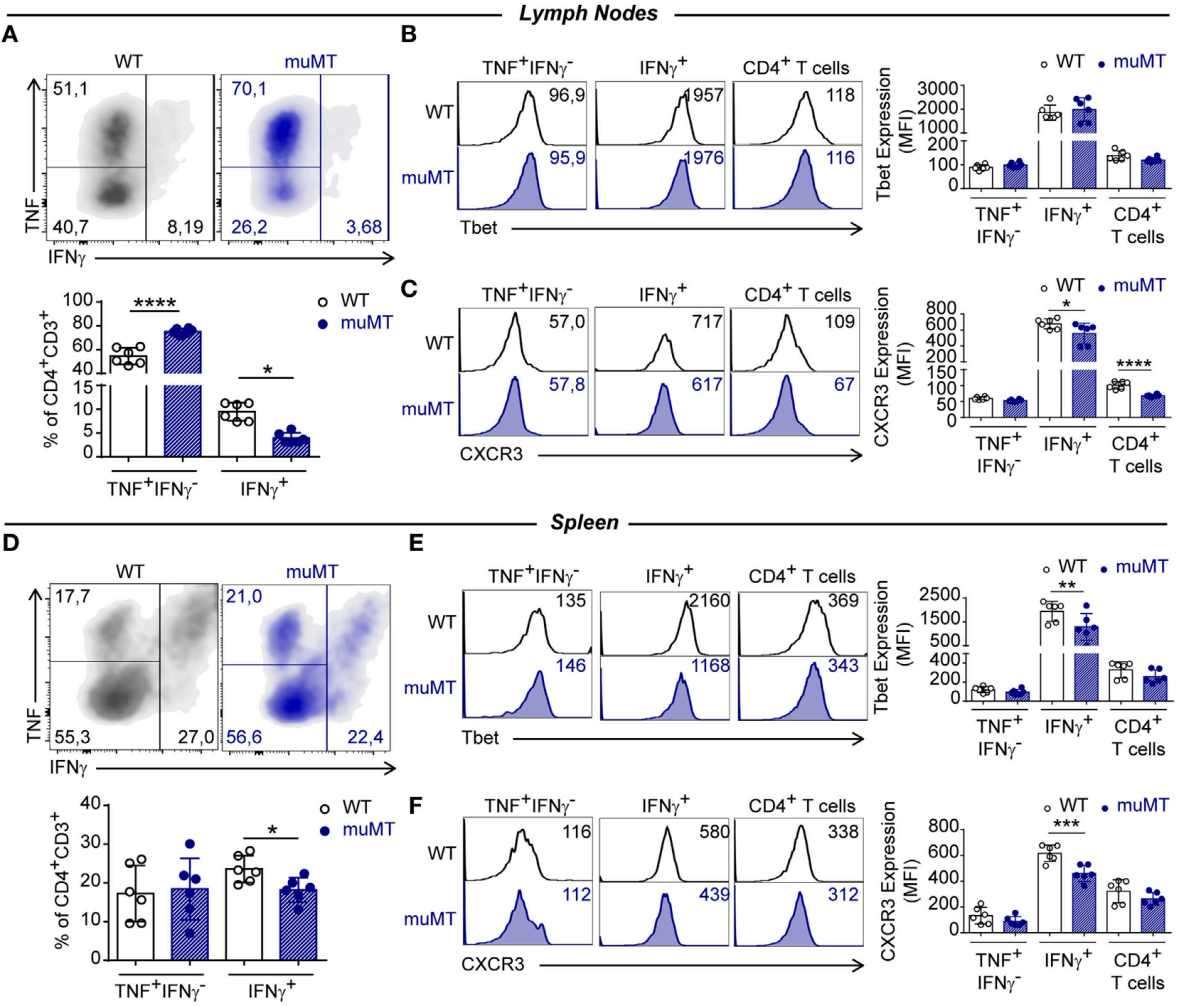
B cell deficiency affected the frequency of IFNγ-producing CD4^+^ T cells in *Trypanosoma cruzi*-infected mice. Representative dot plot showing TNF and IFNγ expression in gated CD4^+^CD3^+^ T cells of **(A)** lymph nodes and **(D)** spleen from infected wild-type (WT) (*n* = 6) and muMT (*n* = 6) mice after 15 days of *T. cruzi* infection; and statistical analysis of the percentage of INF^+^IFNγ^−^CD4^+^CD3^+^, IFNγ^+^CD4^+^CD3^+^, and CD4^+^CD3^+^ T cell subsets. Bars represent mean ± SD of the percentages of each population of 5–6 mice/group. Each symbol represents an individual mouse. Representative histograms and statistical analysis of Tbet and CXCR3 expression on INF^+^IFNγ^−^CD4^+^CD3^+^, IFNγ^+^ CD4^+^CD3^+^, and CD4^+^CD3^+^ T cell subsets from **(B,C)** lymph nodes or **(E,F)** spleen. Bars represent mean ± SD of Tbet or CXCR3 geometric mean in each population (5–6 mice/group). Each symbol represents an individual mouse (*p < 0.05, **p < 0.01, ***p < 0.001, ****p < 0.0001; two-tailed *t*-test). Experiment representative of 3.

In muMT mice, at 15 Dpi, we observed an increase in the percentage of TNF^+^IFNγ^−^-producing cells among the CD4^+^ T cell population in lymph nodes, while IFNγ-producing CD4^+^ T cells in lymph nodes and spleen were significant decreased, in comparison to their WT-infected counterparts (Figures 4A,D, respectively). As expected, IFNγ-producing CD4^+^ T cells presented the highest expression of T-bet (Figures 4B,E) and CXCR3 (Figures 4C,F) in comparison to other populations. However, IFNγ-producing CD4^+^ T cells in the spleen from muMT-infected mice exhibited a significantly lower expression of these molecules than CD4^+^ T cells from infected WT mice. When T-bet and CXCR3 expressions were measured in the total CD4^+^ T cell populations from lymph nodes and spleens of infected muMT and WT mice, we observed these cells presented similar MFIs (Figures 4B,E,F), with the exception of CD4^+^ T cells from the lymph nodes of infected muMT mice which exhibited a significant reduction in CXCR3 expression (Figure 4C).

Interestingly, a significant reduction in the percentages of polyfunctional TNF^+^IFNγ^+^ CD4^+^ T cells was detected in the lymph nodes and spleen from infected muMT mice compared to infected WT (Figures S4A,C in Supplementary Material, respectively). Splenic polyfunctional TNF^+^IFNγ^+^ CD4^+^ T cells from infected muMT mice exhibited a reduction in T-bet and CXCR3 expression, in comparison to their counterparts in infected WT mice; however, this reduction was not observed in lymph nodes (Figure S4B,D in Supplementary Material, respectively).

In addition, it is important to point out that infected muMT mice also presented a significant reduction in IL-17-producing CD4^+^ T cells and no changes in IL-10^+^ CD4^+^ T cells in comparison to infected WT mice (Figure S5 in Supplementary Material). In summary, all data indicate that the absence of B cells altered the polarization of CD4^+^ T cells into Th1-, Th17-, and IL-10-producing CD4^+^ T cells.

### *T. cruzi*-Infected muMT Mice Presented a High Percentage of Unconventional Naïve CD4^+^ T Cells

**Figure 5:**
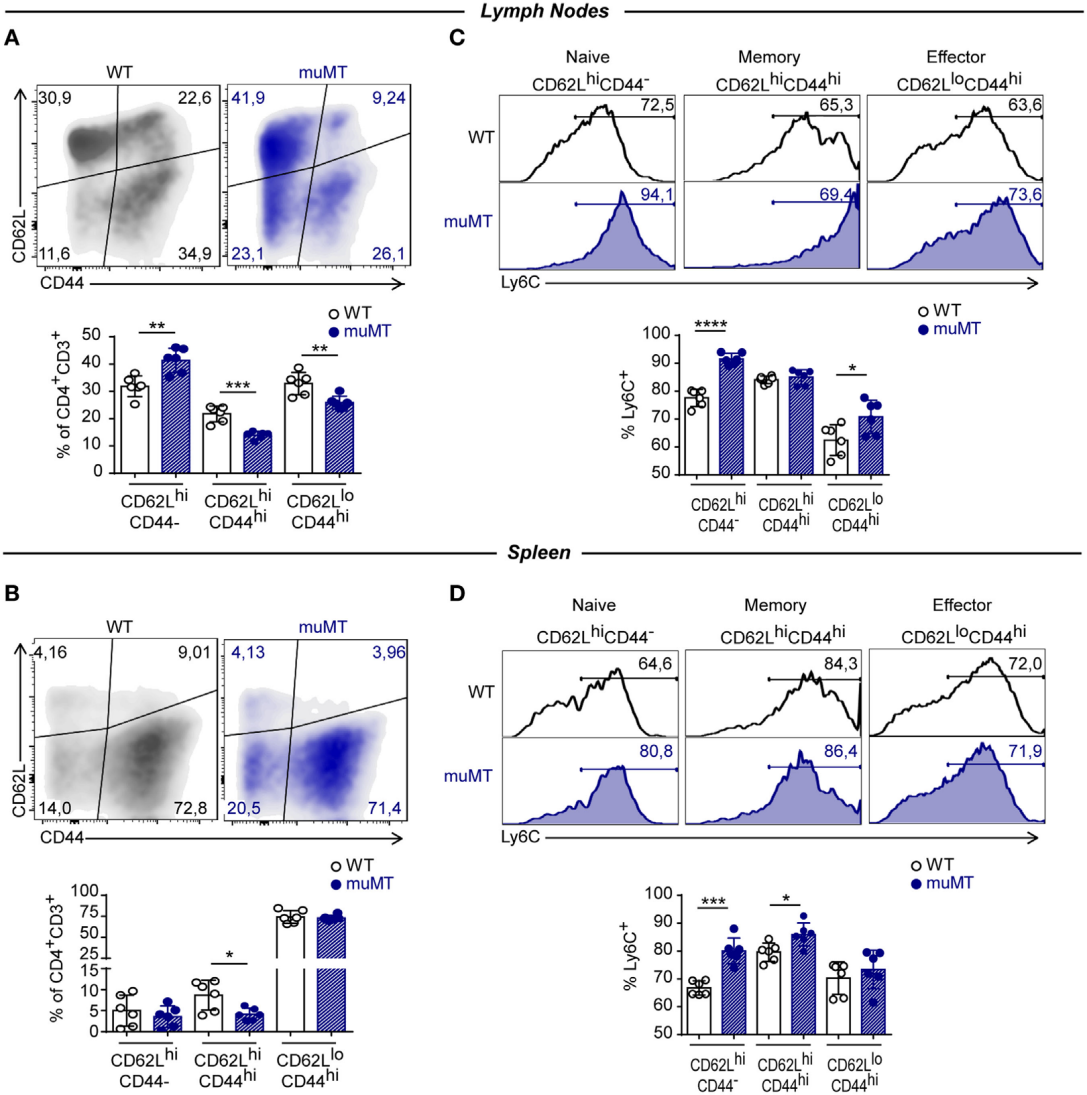
*Trypanosoma cruzi*-infected muMT mice had a high percentage of unconventional naïve CD4^+^ T cells. Representative dot plot showing the expression of CD62L vs. CD44, gated on CD4^+^CD3^+^ T cells in **(A)** lymph nodes and **(B)** spleen from wild-type (WT) (*n* = 5) and muMT (*n* = 6) mice infected with 10,000 trypomastigotes of *T cruzi*; and statistical analysis showing percentages of naïve (CD62^+^CD44^−^), memory (CD62^+^CD44^+^), and effector (CD62^−^CD44^+^) CD4^+^CD3^+^ T cells. Bars represent mean ± SD of the percentage of each population in 5–6 mice/group. Each symbol represents an individual mouse. **(C,D)** Representative histograms showing the percentage and statistical analysis of Ly6C^+^ cells on CD62L^+^CD44^−^, CD62L^+^CD44^+^, and CD62L^−^CD44^+^ CD4^+^CD3^+^ T cells from **(C)** lymph nodes or **(D)** spleen. Bars represent means ± SD of the percentages of Ly6C^+^ cells of each population of 5–6 mice/group. Each symbol represents an individual mouse (*p < 0.05, **p < 0.01, ***p < 0.001, ****p < 0.0001; two-tailed *t*-test). Experiment representative of 3.

It has been reported that TNF is secreted early during the effector response whereas IFNγ is secreted later by effector and differentiated T cells (38). Furthermore, Ly6C has been proposed as a marker for distinguishing short-living effector Th1 cells (Ly6C^hi^) from the memory T-bet^+^CD4^+^ T cells (Ly6C^lo^) (36). Considering these functional and phenotypic attributes that allow to establish different stages during the effector responses together with our results, we hypothesized that infected muMT mice may present altered distribution of naïve, effector, and memory CD4^+^ T cell subpopulations. The assessment of that distribution among CD4^+^ T cells was based on CD62L vs. CD44 expression and showed that CD4^+^ T cells from lymph nodes of infected muMT mice obtained at 15 Dpi presented a higher percentage of naïve (CD62L^hi^CD44^−^) and lower percentages of effector and memory (CD62L^hi^CD44^hi^) cells in comparison to their WT counterparts (Figure 5A). In spleen, infected muMT mice showed only a significant reduction in the frequency of memory CD62L^hi^CD44^hi^CD4^+^ T cells (Figure 5B). A higher percentage of Ly6C^+^ cells was observed within naïve CD4^+^ T cells from spleen and lymph nodes, effector CD4^+^ T cells from lymph nodes and memory CD4^+^ T cells from the spleen of infected muMT mice (Figures 5C,D). Some of these changes were already observed in the spleen at 9 Dpi in which there was an over representation of Ly6C^+^ naïve cells among the total CD4^+^ T cell population at the expense of a decrease of effector and memory CD4^+^ T cell populations (data not shown).

### CD4^+^ T Cells from *T. cruzi*-Infected muMT Mice Exhibit Low Expression of Inhibitory Receptors

Coinhibitory receptors are critical for the maintenance of immune homeostasis. Upregulation of these receptors on effector T cells terminates T cell responses (39). Based on this, and considering than the absence of B cells during *T. cruzi* infection resulted in an exacerbated CD4^+^ T cell response, we evaluated whether CD4^+^ T cells from infected muMT mice showed an altered expression pattern of inhibitory receptors.

**Figure 6:**
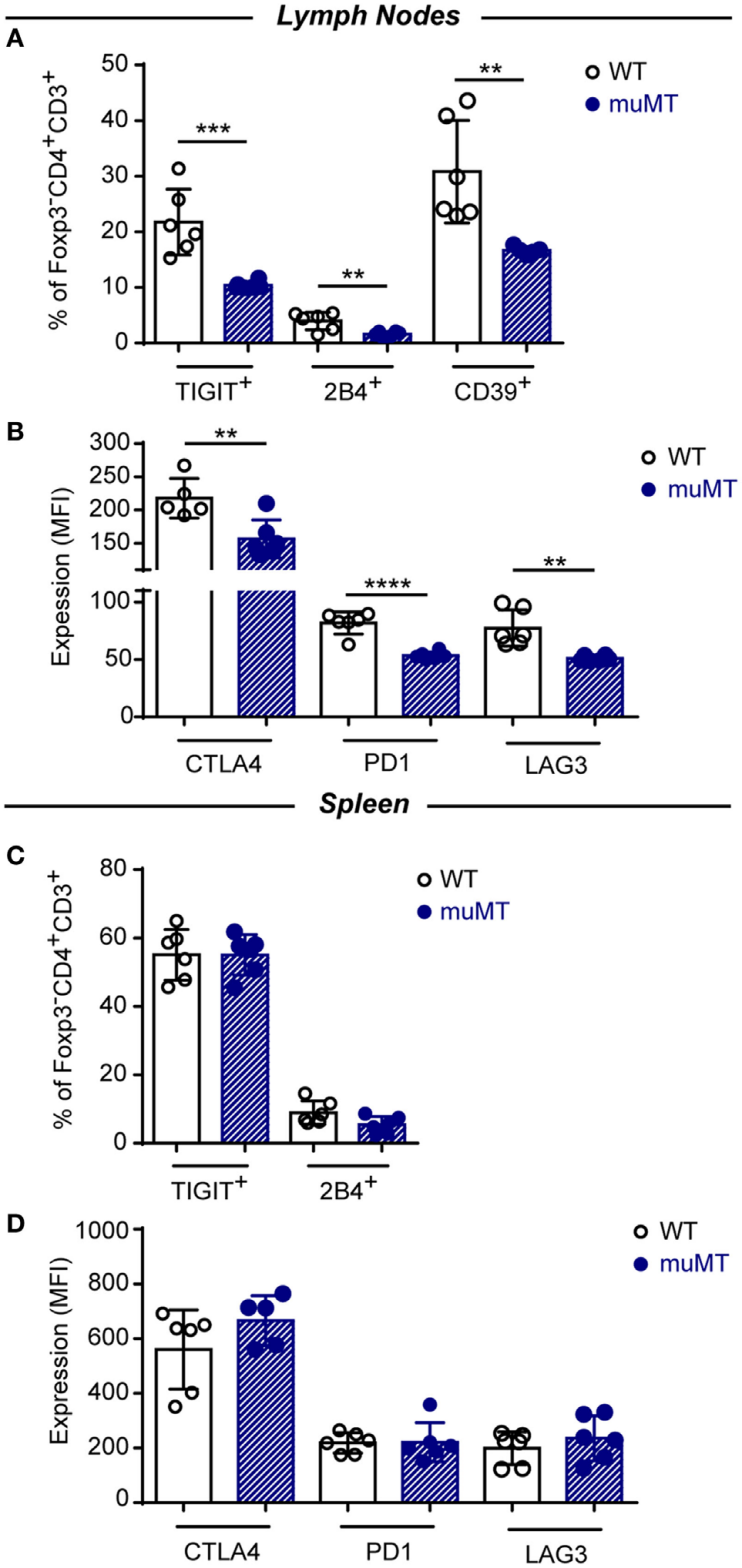
CD4^+^ T cells of infected muMT mice displayed low percentages of cells expressing inhibitory receptors and low expression of inhibitory receptors. **(A,C)** Percentage of TIGIT and 2B4 expressing cells in gated Foxp3^−^CD4^+^CD3^+^ T cells in **(A)** lymph nodes and **(C)** spleen from wild-type (WT) (*n* = 6) and muMT (*n* = 6) mice infected with 10,000 trypomastigotes of *Trypanosoma cruzi* obtained at 15 days post infection (Dpi). **(B,D)** Expression (MFI) of CTLA4, PD1, and LAG3 in gated Foxp3^−^CD4^+^CD3^+^ T cells in **(B)** lymph nodes and **(D)** spleen from infected WT and muMT mice obtained at 15 Dpi. Bars represent means ± SD of the percentage of cells expressing the inhibitory receptor or inhibitory receptor expression on Foxp3^−^CD4^+^CD3^+^ T cells. Each symbol represents an individual mouse (**p < 0.01, ***p < 0.001, ****p < 0.0001; two-tailed *t*-test). Experiment representative of 2.

In Figure 6, we depicted the percentage of cells expressing the inhibitory receptor when inhibitory molecules presented bimodal distribution as TIGIT and 2B4, and intensity of expression for molecules that presented unimodal distribution as CTLA-4, PD-1, and LAG-3. Figure 6 shows that the percentage of TIGIT^+^, 2B4^+^, and CD39^+^ conventional Foxp3^−^CD4^+^ T cells (Figure 6A) and the expression of CTLA-4, PD-1, and LAG3 on these cells (Figure 6B) were significantly reduced in the lymph nodes of infected muMT mice, in comparison to their counterpart from infected WT mice. No significant changes were observed between these populations in the spleens from both experimental groups (Figures 6C,D).

The lower MFI values for CTLA4, PD1, and LAG3 expression on CD4^+^ T cells from infected muMT mice could simply be due to the lower percentage of CD44^+^ CD4^+^ T cells in the lymph nodes and spleen of muMT mice. Therefore, activated CD4^+^ T cells could be diluted by “non-activated CD4^+^ T cells” which results in a lower MFI in the population. Then, we evaluated the expression of CTLA-4, PD-1, and LAG-3 on CD44^+^ CD4^+^ T cells and we observed a significant reduction in CTLA-4 and PD-1 expression on activated CD44^+^ CD4^+^ T cells from lymph nodes and spleen of infected muMT mice (Figure S6 in Supplementary Material). Then, the lower MFI values for CTLA4 and PD1 observed on CD4^+^ T cells from muMT mice were not due to a lower percentage of activated CD4^+^ T cells.

### Infected muMT Mice Had Low Percentage of Foxp3^+^ Tregs

In recent years, it has been recognized that acquired immunity is controlled by Tregs (40, 41) and this activity may be exerted directly thought different surface molecules such as CTLA-4 (42), PD-1 (43), LAG-3 (44), and CD39 (45). Given that absence of B cells in *T. cruzi* infection correlated with an exacerbated CD4^+^ T cell effector response, we hypothesized that Tregs may be affected in this experimental setting.

**Figure 7:**
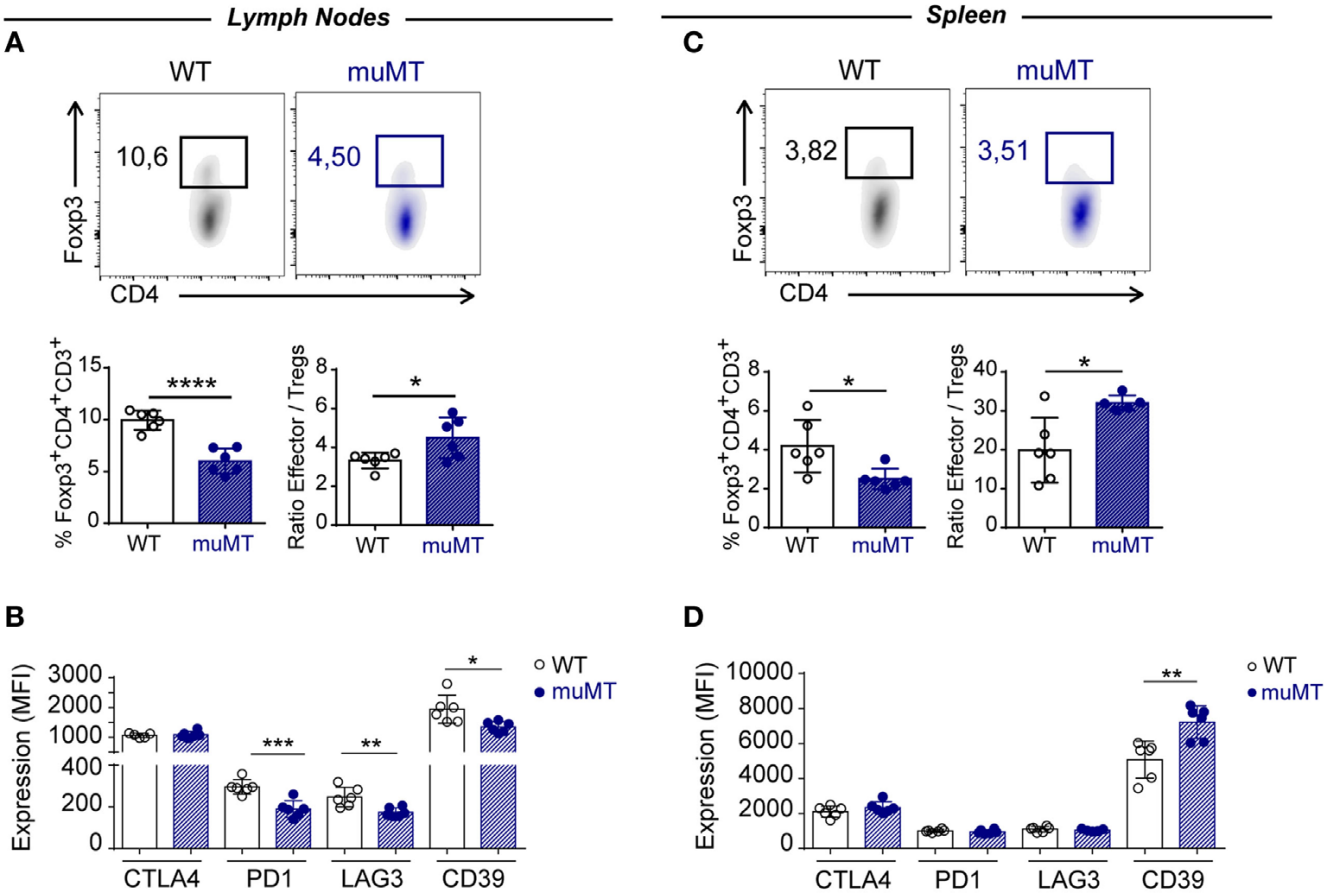
Infected muMT mice exhibit a low percentage of Foxp3^+^ Tregs. **(A,C)** Representative dot plots of the percentage of Foxp3^+^ cells gated on CD4^+^CD3^+^ T cells of **(A)** lymph nodes and **(C)** spleen from wild-type (WT) (*n* = 6) and muMT (*n* = 6) infected with 10,000 trypomastigotes of *Trypanosoma cruzi*, obtained at 15 days post infection (Dpi). Bars represent mean ± SD of the percentage of Foxp3^+^CD4^+^CD3^+^ T cells and the ratio between effectors CD4^+^ CD3^+^ T cells and Foxp3^+^ Tregs. **(B,D)** Expression of CTLA4, PD1, LAG3, and CD39 on Foxp3^+^CD4^+^CD3^+^ T cells of **(B)** lymph nodes and **(D)** spleen from infected WT and muMT mice obtained at 15 Dpi. Bars represent mean ± SD of inhibitory receptor expression on Foxp3^+^ Tregs, determined by geometric mean. Each symbol represents an individual mouse (*p < 0.05, **p < 0.01, ***p < 0.001, ****p < 0.0001; two-tailed *t*-test).

Analysis of Foxp3^+^CD4^+^CD3^+^ T cells showed that infected muMT mice presented a significantly reduced percentage of these cells in the lymph nodes and spleen at 15 Dpi when compared to infected WT mice (Figures 7A,C). Of note, the ratio between effector T cells and Foxp3^+^ Tregs cells was higher in infected muMT mice (Figures 7A,C). Absence of B cells also affected the expression of the surface mediators PD-1, LAG3, and CD39 on Foxp3^+^ Tregs from lymph nodes (Figure 7B) but no changes in the expression of these molecules (except for CD39) were observed on splenic Tregs from muMT and WT mice (Figure 7D).

### Unconventional CD4^+^ T Cells from muMT Mice Can Modulate the Profile of CD4^+^ T Cells despite the Presence of B Cells

**Figure 8:**
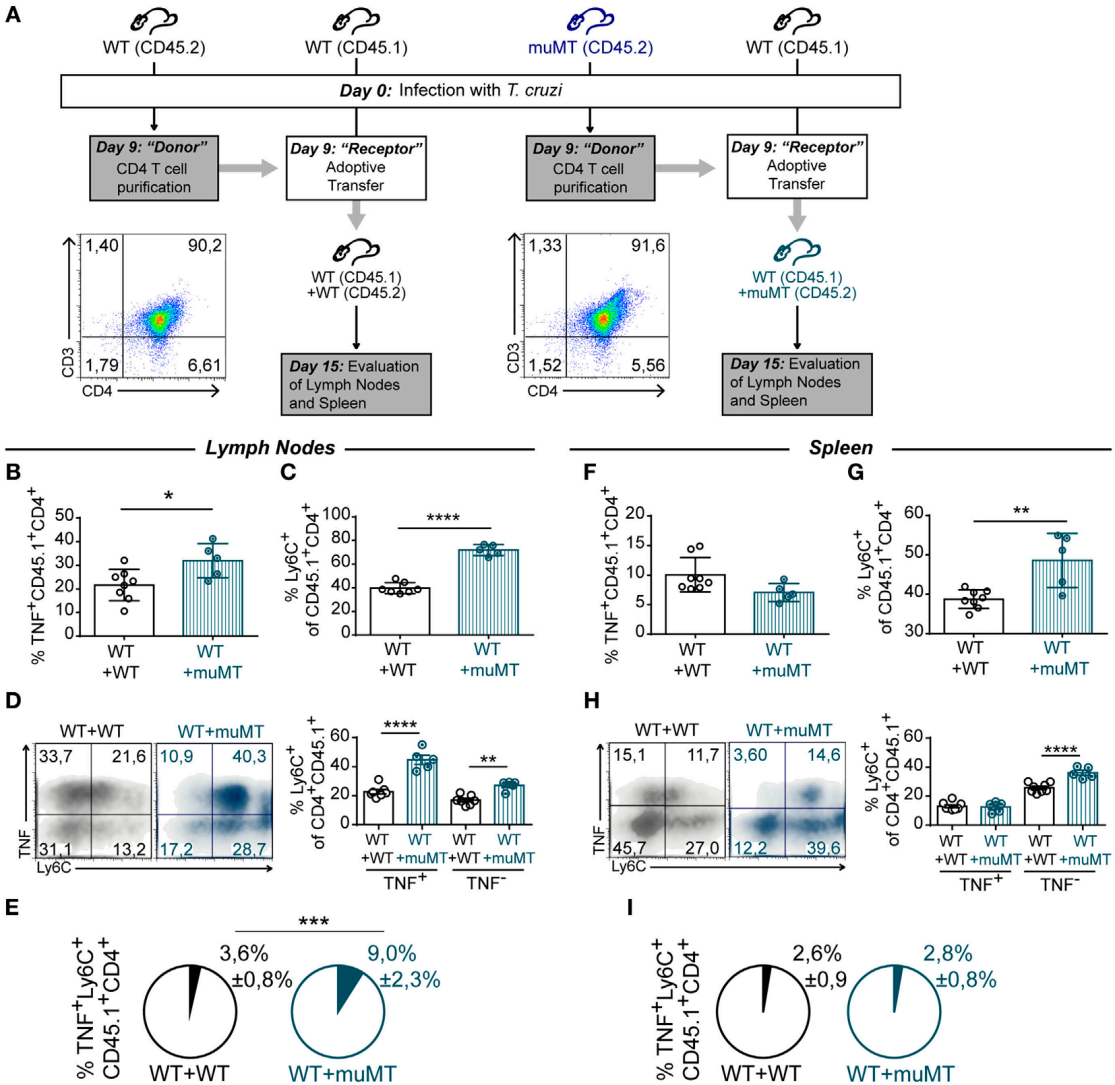
Unconventional T cells from muMT mice influence the profile of CD4^+^ T cell from wild-type (WT) mice. **(A)** Outline of the experimental design. WT (*n* = 11) and muMT (*n* = 8) mice were infected with 10,000 trypomastigotes of *Trypanosoma cruzi* Y strain (day 0pi). At 9 days post infection (Dpi), splenic CD4^+^CD3^+^ T cells were obtained from infected WT and muMT mice and transferred to infection-matched WT CD45.1 mice. These mice were named WT + WT or WT + muMT, respectively. At 15 Dpi, all mice were culled WT + WT (*n* = 7) and WT + muMT (*n* = 5) and TNF^+^ and Ly6C^+^ cells in CD45.1 CD4^+^ T cells were evaluated. Dot plots shows the purity of transferred CD4^+^ T cells. **(B,F)** Statistical analysis of the percentage of TNF^+^CD45.1^+^CD4^+^ T cells in lymph nodes and spleen of WT + WT and WT + muMT mice at 15 Dpi, respectively. **(C,G)** Statistical analysis of the percentage of Ly6C^+^ cells on CD45.1^+^CD4^+^CD3^+^ T cells in lymph nodes and spleen at day 15 Dpi, respectively. **(D,H)** Representative dot plot showing TNF and Ly6C expression in gated CD45.1^+^CD4^+^CD3^+^ T cells in lymph nodes and spleen, and statistical analysis of the percentage of Ly6C^+^ cells in TNF^−^ and TNF^+^CD4^+^CD3^+^ T cells. Bars represent means ± SD of the percentage. **(E)** Pie graphs showing the mean proportion ± SD of TNF^+^Ly6C^+^CD45.1^+^CD4^+^CD3^+^ T cells respect to the total cell number in lymph nodes **(E)** and spleen **(I)** (*p < 0.05, **p < 0.01, ****p < 0.0001; two-tailed *t*-test).

During *T. cruzi* infection, B cell deficiency results in CD4^+^ T cells acquiring an unconventional profile that may be pathogenic. Then, we next evaluated whether CD4^+^ T cells from muMT mice can influence the CD4^+^ T cell response of infected WT mice, turning it into a more inflammatory phenotype. For that, muMT (CD45.2) and WT (CD45.2 and CD45.1) mice were infected and, at 9 Dpi, CD4^+^ T cells were purified from the spleen of CD45.2 WT or CD45.2 muMT mice and adoptively transferred to infection-matched CD45.1 WT mice as schematized in Figure 8A (WT + WT and WT + muMT, respectively). After 15 Dpi, we determined the frequency of TNF^+^ CD45.1^+^CD4^+^ T cells in the recipient mice. We observed that, at 15 Dpi, WT mice that received CD4^+^ T cells from muMT mice presented a higher percentage of TNF^+^ CD45.1^+^CD4^+^ T cells in lymph nodes (Figure 8B, WT + muMT), but not in the spleen (Figure 8F), in comparison to WT mice that received CD4^+^ T cells from infected WT mice (Figure 8B, WT + WT). We next evaluated the percentage of Ly6C^+^ cells among the CD45.1^+^CD4^+^ T cell population, and observed that WT + muMT mice showed a significantly increased percentage of Ly6C^+^ cells in the endogenous CD45.1^+^CD4^+^ T cells in lymph nodes and spleen (Figures 8C and **G**). To evaluate whether there had been a change in functionality of endogenous CD4^+^ T cells due to the injection of unconventional CD4^+^ T cells from muMT, we evaluated the expression of Ly6C and TNF in CD45.1^+^CD4^+^ T cells. We observed that WT + muMT mice presented a higher percentage of Ly6C^+^TNF^−^ and Ly6C^+^TNF^+^ CD4^+^ T cells in lymph nodes (Figure 8D), and an increase in Ly6C^+^TNF^+^CD4^+^ T cells in spleen (Figure 8H) at 15 Dpi within the endogenous CD45.1^+^ CD4^+^ T cells. The increase in the percentage of Ly6C^+^TNF^+^ cells among the CD45.1^+^CD4^+^ T cell population in lymph nodes represented an increase in the frequency of Ly6C^+^TNF^+^CD4^+^ T cells also among all leukocytes in lymph nodes (Figure 8E, see Pie chart), but not in the spleen (Figure 8I, see Pie chart).

### Plasmablast/Plasma Cell-Deficient Mice Infected with *T. cruzi* Exhibited High Parasitemia and Frequency of TNF-Producing CD4^+^ T Cells

**Figure 9:**
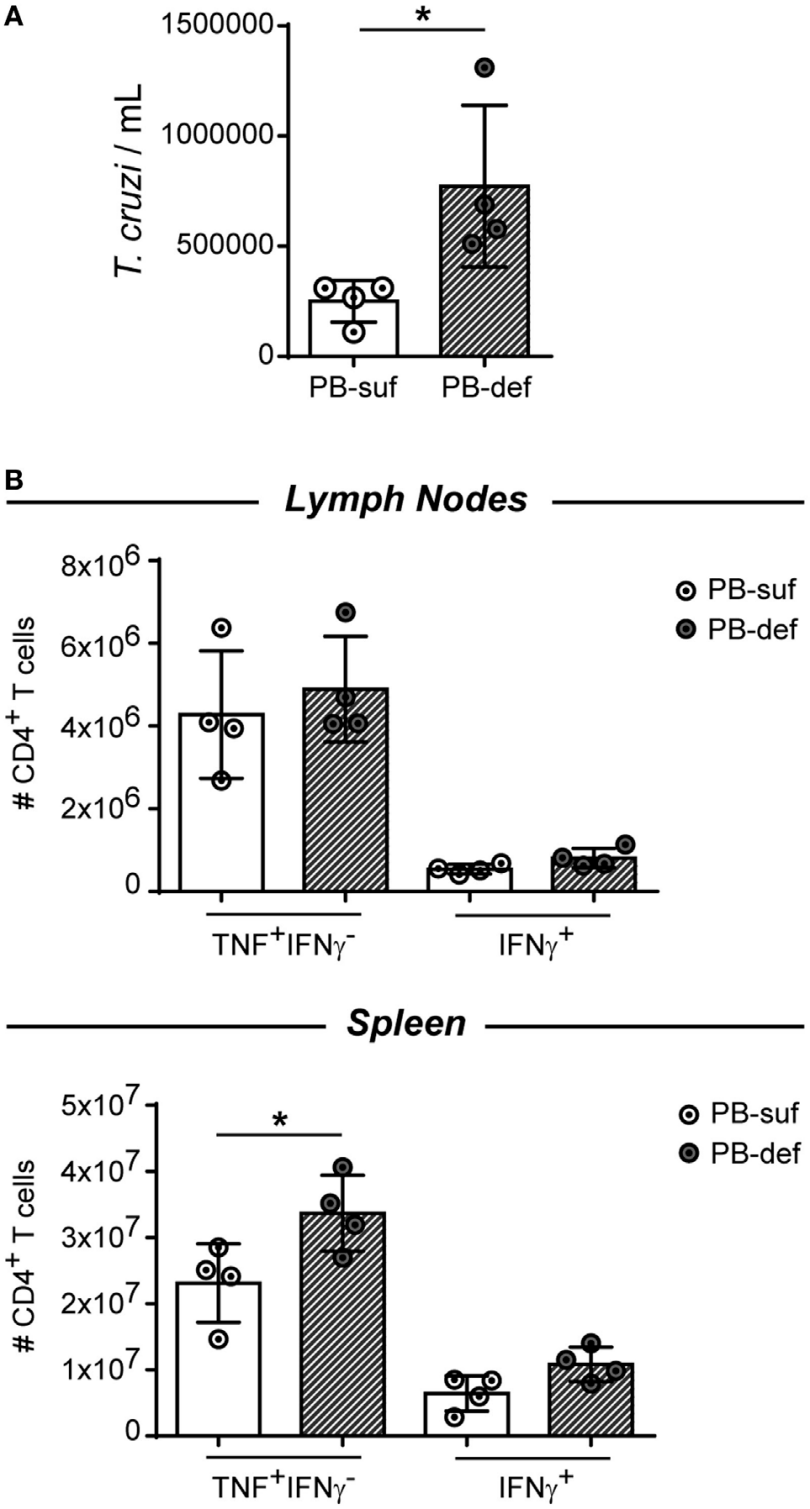
Plasmablast/plasma cell-deficient mice infected with *Trypanosoma cruzi* exhibited a significant number of trypomastigotes in blood and splenic TNF^+^IFNγ^−^CD4^+^ T cells. CD23icre (*n* = 4, empty circles) and Blimp-flox/flox-CD23icre mice (*n* = 4, fill circles) mice were infected with 10,000 trypomastigotes of *T. cruzi* Y strain. CD23icre mice were indicated as plasmablast sufficient mice (PB-suf) and Blimp-flox/flox-CD23icre mice were indicated as PB-deficient mice (PB-def). Mice were bleed at 9 Dpi to determine parasitemia and were culled at 15 days post infection (Dpi) and lymph nodes and spleens were obtained for flow cytometry analysis. **(A)** Number of trypomastigotes in blood samples at 15 Dpi. **(B)** Number of TNF^+^ CD4^+^ CD3^+^ T cells in lymph nodes or spleen of infected mice.

To answer the question about the role of antibodies in these model, we infected Blimp1ff/CD23cre mice, which are deficient in plasmablasts and plasma cells and consequently in antibodies. These mice will show us whether plasmablasts and plasma cells and antibody production, independently of antigen-presenting B cells and B-cell-derived cytokines, are involved in the parasite control and/or regulates the inflammatory response. Figure 9 shows that Blimp1ff/CD23cre mice infected with *T. cruzi* exhibit a significant higher number of trypomastigotes in blood (Figure 9A) and splenic TNF^+^IFNγ-CD4^+^ T cells in comparison to infected CD23cre mice (Figure 9B), indicating that plasmablasts and plasma cells are important for parasite replication control and suggest that plasmablasts and plasma cells could exert a regulatory role controlling TNF-producing cells. Interestingly, the number of Foxp3^+^ CD4^+^ T cells was similar between infected Blimp1ff/CD23cre mice and their controls (data not shown).

## Discussion

During the past decades, many reports have demonstrated that B cells, beyond being the only Ab-producing cells, can influence immunity in multiple ways such as antigen presentation to T cells, expression of surface co-stimulatory/inhibitory molecules and cytokine secretion. Consequently, B cells can act as drivers of innate and adaptive immunity (46). In this report, we demonstrate that in *T. cruzi* infection the absence of mature B cells affects the CD4^+^ T cell subsets response. Our findings show that B cells play a key role in shaping CD4^+^ T cells profile since the absence of B cells leads to a reduction in the frequency of Th1, Th17, and Foxp3^+^ Treg cells concomitant with an increase in the frequency of TNF-producing CD4^+^ T cells. Consequently, *T. cruzi*-infected muMT mice exhibit higher concentrations of TNF in plasma.

Cardillo et al. (31) reported that muMT mice exhibit an intensified infection with *T. cruzi* probably due to an insufficient development of an inflammatory T cell response, particularly CD8^+^. On the other hand, we have observed that infected muMT mice are more susceptible to the infection, probably due to immunopathological mediators, since parasitemia and parasite loads can be controlled in infected muMT mice, but in the presence of exacerbated levels of inflammatory cytokines. Probably the differences between our results and that previous report are due to the different strains of *T. cruzi* used. Cardillo et al. (31) have used *Tulahuen* strain of *T. cruzi*, which is more virulent than the Y strain used in our work.

TNF has been described long ago as a key cytokine-mediating protection in *T. cruzi* infection as it has been proven to be necessary for parasite clearance in blood and for inducing effective tissue inflammation for parasite load control (47). Its protective role has been assigned to the amplification of NO production (48–50) and to the induction of a germinal center response with generation of specific Abs for *T. cruzi* (51). Despite its well-established role in protection during *T. cruzi* infection, TNF has also been described as a mediator of immunopathology (33, 52). We determined that high levels of plasma TNF in *T. cruzi*-infected muMT mice were associated with increased liver pathology, decreased body weight, increased liver transaminases, altered kinetic of plasma glucose, and high mortality.

There have been a few reports showing an increase in the TNF response, in absence of B cells, during infections. In *Toxoplasma gondii* infection, lack of B cells led to increased mRNA for TNF in brain which was associated with increased *T. gondii* replication (53). Significantly increased numbers of *T. gondii* cysts and areas of inflammation associated with tachyzoites were observed in the brains of infected muMT mice compared to control mice, and these were prevented by the administration of anti-*T. gondii* IgG. Unfortunately, the levels of mRNA for TNF were not determined after Ab treatment so the control of parasite replication may be associated with Ab production, although it remains unclear whether TNF was contributing to parasite control or inflammation (53). A similar phenomenon was described by Onyilagha and collaborators who determined that the deficiency of the B cell adaptor molecule Bam32 causes significantly higher amounts of TNF and other pro-inflammatory cytokines such as IFNγ and IL6-produced by CD4^+^ T cells, and results in a failure to control parasitemia during the chronic phase of *Trypanosoma congolense* infection (54). Thus far, in *T. gondii* and *T. congolense* infections, increased TNF production is concomitant with high parasite load, contrary to our work in which high TNF levels were present with low parasite replication and associated to tissue damage. In our model, we observed that, at initial Dpi, *T. cruzi*-infected muMT mice presented higher parasitemia and parasite load in comparison to infected WT mice; however, during the late acute phase of the infection, the parasitemia and parasite load were similar to those presented by infected WT mice, suggesting that despite the lack of antibodies, infected muMT mice were able to control parasite replication and this is not the cause for the exacerbation of the TNF inflammatory response. Our findings suggest that beyond Ab production, B cells can influence TNF responses probably through cytokine secretion since we reported that during *T. cruzi* infection, B cells secrete IL-17 and, *via* this cytokine, B cells regulate TNF levels in muMT-infected mice. B220^+^IL-17^+^ cells were undetectable in *T. cruzi*-infected muMT mice and the development of this population was restored in recipients of adoptively transferred mature B cells. IL-17^+^ cells induced in B cell-reconstituted infected muMT mice had a surface phenotype indistinguishable from that observed in infected WT mice (B220^dim^CD19^+^CD138^+^GL7^−^ PNA^−^IL-17^+^ cells) indicating that IL-17-secreting cells exhibit a plasmablast phenotype. Plasmablasts from *T. cruzi*-infected mice secrete IL-17, control parasitemia, and decrease plasma TNF levels in muMT-infected mice, since muMT recipients of IL-17A-deficient B cells had higher parasitemia and high levels of TNF compared to muMT recipients of WT B cells. IL-17-producing B cells compose a major source of this cytokine, outnumbering Th17 cells. Thus, B cells were the main producers of IL-17A during the acute infection with *T. cruzi*, and B cell-intrinsic production of IL-17A was critical for the protective response to this pathogen (28). In addition, we observed that B cells from *T. cruzi*-infected mice produced many cytokines such as IL-6, IL-10, and TGF-β (data not shown), suggesting that beyond IL-17, other cytokines produced by B cells can influence different levels of immunity. The experiments performed with Blimp1ff/CD23cre mice reinforce the idea that plasmablast/plasma cell, beyond producing antibodies, act as regulatory cells.

In *T. cruzi*-infected muMT mice, TNF-producing cells are mostly CD4^+^ T cells, implying that B cells condition the CD4^+^ T cell response during the infection. Moreover, *T. cruzi*-infected muMT mice exhibited a high frequency of Ly6C^+^TNF-producing CD4^+^ T cells. Ly6C expression has been associated to a Th1 pro-inflammatory profile (36, 37), but interestingly, mature B cell absence in *T. cruzi* infection did not favor the polarization of CD4^+^ T cells to a Th1. We observed that at the peak of parasitemia, muMT-infected mice presented higher frequency of IFNγ-producing CD4^+^ T cells than their counterparts in infected WT, but this population rapidly decreased (data not shown). In fact, CD4^+^ T cells from 15 days-*T. cruzi*-infected muMT mice presented a diminished percentage of IFNγ-producing cells, as well as reduced T-bet and CXCR3 expression. It has been reported that differential expression of Ly6C and T-bet on virus-specific CD4^+^ T cells distinguishes effector from memory Th1 CD4^+^ cells (36). Ly6C^lo^CD4^+^ T cells were reported to provide the effector and memory T cells, while Ly6C^hi^CD4^+^ T cells are effector short-lived cells (36, 55). Taking this information into account, it may be possible to say that in absence of B cells, short-lived effector CD4^+^ T cells are generated during *T. cruzi* infection; however, these reports assessed the specific CD4^+^ T cell population, whereas we evaluated the whole CD4^+^ T cell compartment.

Interestingly, there is no difference in the percentages of TNF^+^Ly6C^+^CD4^+^ T cells in the spleen of infected mice at 15 Dpi but at this time point, muMT mice exhibit a higher parasite load in the spleen respect to WT. This observation reinforces the idea that this TNF^+^ population may not be involved in parasite control, but might have a deleterious effect. Considering that TNF^+^Ly6C^+^CD4^+^ T cells may be regulated by cell populations that are diminished or absents in *T. cruzi*-infected muMT, it is possible that its presence has a greater deleterious effect in the absence of B cells or their product of differentiation.

Based on the expression of CD44 and CD62L on CD4^+^ T cells, we observed that a lower frequency of lymph nodes CD4^+^ T cells from infected muMT mice acquired effector and memory phenotypes and a higher percentage of CD4^+^ T cells remains in a “naïve” state. This result suggests that a higher percentage of CD4^+^ T cells activated during *T. cruzi* infection in absence of B cells are non-antigen experienced (56) but as they produce TNF and express Ly6C, they are probably activated through bystander signals. This is consistent with the notion that during this parasite activation the CD4^+^ T cell pool comprises both parasite-specific cells and non-specific cell that underwent polyclonal activation (57). In addition, as reported in mice without mature B cells infected with LCMV (55), *T. cruzi*-infected muMT mice exhibit a decrease in the frequency of memory CD4^+^ T cells, suggesting that B cells can also sustain memory T cells during *T. cruzi* infection.

It is well known that TNF has the capacity to promote TCR-dependent T cell activation as well as to activate Tregs (58); however, TNF action on naïve, effector, and memory CD4^+^ T cell subpopulations was not evaluated. It has been reported that mice deficient in TNFR1 infected with *T. cruzi* do not present any changes in the frequency of effector CD44^+^CD62L^low^CD8^+^ T cells (59), and although this has not been reported for CD4^+^ T cells, we can speculate that the decreased frequencies of effector and Foxp3^+^ CD4^+^ T cells in lymph node from infected muMT mice are not consequence of high TNF signaling. The differential proportions of naïve, effector, and memory CD4^+^ T cells present in infected muMT mice, in comparison to infected WT, can be attributed to B cell-antigen presentation. It has been reported that B cells are involved in a MCH-II-dependent priming of CD4^+^ T cells to promote CD4^+^ T cell activation and differentiation into effector and memory CD4^+^ T cells producing IFNγ, IL2, and TNF (60).

The unconventional profile of TNF-producing CD4^+^ T cells from infected muMT mice was accompanied by a reduced expression of inhibitory receptors. The immune regulation by the host seems to be an essential factor for Chagas disease progression, and immune system inhibitory molecules such as PD-1 and CTLA-4 favor the maintenance of peripheral tolerance (61, 62). The expression of CTLA-4, PD-1, and LAG3 on CD4^+^ T cells and the percentage of TIGIT^+^, 2B4^+^, and CD39^+^ conventional Foxp3^−^ CD4^+^ T cells in infected muMT mice were significantly reduced, suggesting that, in absence of B cells, *T. cruzi*-infected mice presented CD4^+^ T cells that potentially could be less regulated. PD-1 signaling has been shown to be a very important regulatory pathway during *T. cruzi* infection since Gutierrez et al. demonstrated that lack of signaling through the PD-1 pathway (using a blocking Ab or PD-1KO mice) in infected mice leads to decreased parasitemia and parasite load in tissues, but to increased mortality due to an overexpression of the pro-inflammatory Th1 response (62). CTLA4 is also important in the regulation of the pro-inflammatory Th1 response in *T. cruzi* infection and *in vivo* and *in vitro* blockade of CTLA4 enhanced IFNγ and especially TNF secretion by splenocytes cultured with parasite antigens (61). In humans, polymorphisms at the CTLA4 gene may determine the symptoms during the chronic phase of Chagas disease; it has been shown that an increase in the expression of CTLA-4 was associated with the indeterminate form of the disease (63). In addition, LAG-3 has been described also as a coinhibitory receptor repressing the T cell response in autoimmunity and cancer (39). Accordingly, we found a decrease in LAG-3 expression on CD4^+^ T cells suggesting that, in absence of B cells, CD4^+^ T cells can be primed and become over-reactive during *T. cruzi* infection. Here, we show that the absence of B cells during *T. cruzi* infection modulates the expression of inhibitory receptors, favoring the emergency of effector response able to control parasite replication after the peak of parasitemia but inducing pathology in the host.

The absence of B cells in *T. cruzi*-infected mice also affects the frequency of Foxp3^+^ Tregs. This result agrees with previous reports showing that B-cell-deficient muMT mice have decreased percentages of Treg in periphery (64). It has been reported that resting B cells are able to expand and maintain Tregs (64). Interestingly, Treg cells from *T. cruzi*-infected WT mice showed a reduced proliferation rate and, consequently, their frequency is progressively reduced along infection compared to effector T cells (65). Thus, lack of B cells affect the number of Foxp3^+^ Tregs; and consequently, the ratio effector/Treg cells was increased in infected muMT mice suggesting that in absence of B cells there was a greater expansion of effector CD4^+^ T cells.

It is well established that Foxp3 is a critical transcription factor for development and function of Tregs and a dominant component of their etiology. It has been recently demonstrated that a specific Treg epigenome (a combination of epigenetic marks in Treg-specific genes) is required, in addition to Foxp3, for Treg development, stability, and full effector function (66). Thus, some CD4^+^ T cells may transiently express Foxp3 but will not become bona fide Tregs with regulatory properties unless a Treg epigenome is also acquired. These cells, which would represent a very minor percentage among Foxp3 regulatory T cells, are considered unstable Tregs that loose Foxp3 expression under certain inflammatory environment and acquire effector functions (67). Given this, it is likely that the reduced pool of Foxp3^+^ cells present in the pro-inflammatory environment of infected muMT mice may include unstable non-regulatory Tregs that are in a process of losing Foxp3 expression, turning into effector cells.

Absence of B cells also influences the characteristics of Foxp3^+^ Tregs. The phenotype of Foxp3^+^ Tregs in infected muMT mice was characterized by a lower expression in two molecules involved in immunosuppressive function such as LAG-3 (44) and CD39 (68). It has been reported that antibodies specific to LAG-3 inhibit Treg-mediated suppression both *in vitro* and *in vivo*. Natural CD4^+^CD25^+^ Tregs express LAG-3 upon activation, which is significantly enhanced in the presence of effector cells, whereas CD4^+^CD25^+^ Tregs from LAG-3^−/−^ mice exhibit reduced regulatory activity (44). The CD39/CD73 pathway has gained increasing interest due to its dual role in the control of inflammation *in vivo* (69). Indeed, the ectoenzymes hydrolyze ATP and generate adenosine, thereby converting a pro-inflammatory stimulus into an anti-inflammatory mediator. Francois et al. (70) reported that CD73 expression was downregulated during acute infection with *T. gondii*, leading to impaired capacity to produce adenosine and therefore causing intestinal immunopathology (70). Probably, Foxp3^+^ Tregs from infected muMT mice could present a reduced regulatory function because their reduced expression of suppressive molecules.

Interestingly, in infected Blimp1ff/CD23cre mice, the number of Foxp3^+^ Tregs is non-affected, suggesting that B cell in its role of antigen-presenting cells could influence the characteristic of Tregs more than in its role of antibody-secreting cell or regulatory cell.

Finally, it has been reported that T cells from infected *T. cruzi* mice transferred into uninfected healthy mice infiltrate target organs and induce disease (71). Therefore, we intended to see whether CD4^+^ T cells generated in absence of B cells preserved their phenotype once transferred into a host that was ongoing a concomitant infection and was sufficient in B cells. After the transfer, we observed that the phenotype and function of the T cells from the receptor mice were altered. We found that there was an increase in the total percentage of TNF-producing cells, in lymph nodes and an increase in the percentage of Ly6C^+^CD4^+^ T cells, particularly in the proportion of CD4^+^ Ly6C^hi^ TNF-producing cells in the whole organ despite the presence of B cells.

Summarizing, all the data analyzed together show that absence of B cells and of their product of differentiation as plasmablast/plasma cells conditions the profile of CD4^+^ T cells creating a scenario that favors the induction and persistence of an inflammatory response leading to the death of animals infected with *T. cruzi*.

## Ethics Statement

All animal experiments were approved by and conducted in accordance with guidelines of the committee for Animal Care and Use of the Facultad de Ciencias Quimicas, Universidad Nacional de Cordoba (Approval Number HCD 1525/14) in strict accordance with the recommendation of the Guide to the Care and Use of Experimental Animals published by the Canadian Council on Animal Care (OLAW Assurance number A5802-01).

## Author Contributions

MS performed most of the experiments, analyzed data, prepared figures, and collaborated in manuscript writing. JB, FV, CB, MR, DB, and AC collaborated with experiment performance. CM, ER, and CV contributed to study design, analysis of results, and corrected the manuscript. AG conceived, designed, and supervised the study and wrote the manuscript. All authors reviewed the manuscript before submission.

## Conflict of Interest Statement

The authors declare that the research was conducted in the absence of any commercial or financial relationships that could be construed as a potential conflict of interest.
