# Supplementary material for: Unconventional Pro-inflammatory CD4+ T Cell Response in B Cell-Deficient Mice Infected with Trypanosoma cruzi
Source: Front Immunol. 2017 Nov 21;8:1548. doi: 10.3389/fimmu.2017.01548 (PMC5702327; doi:10.3389/fimmu.2017.01548)
Supplement: Supplementary file 1 [file data_sheet_1.pdf]

Supplementary Material

Unconventional pro-inflammatory CD4+ T cell response in B cell deficient mice infected with *Trypanosoma cruzi*

Melisa Gorosito Serrán[1], Jimena Tosello Boari[1], Facundo Fiocca Vernengo[1], Cristian G Beccaría[1#], María C Ramello[1#], Daniela A Bermejo[1], Amelia G Cook[2], Carola G Vinuesa[2], Carolina L Montes[1], Eva V Acosta Rodriguez[1], Adriana Gruppi[1*].

[1]Centro de Investigaciones en Bioquímica Clínica e Inmunología (CIBICI - CONICET), Departamento de Bioquímica Clínica, Facultad de Ciencias Químicas, Universidad Nacional de Córdoba, Córdoba, Argentina.

[2] Dept of Immunology and Infectious Disease, John Curtin School of Medical Research, Australian National University, Canberra, Australia.

*** Correspondence:** Corresponding Author: Dr Adriana Gruppi. agruppi@fcq.unc.edu.ar

1 Supplementary Figures and Tables

1.1 Supplementary Figure 1

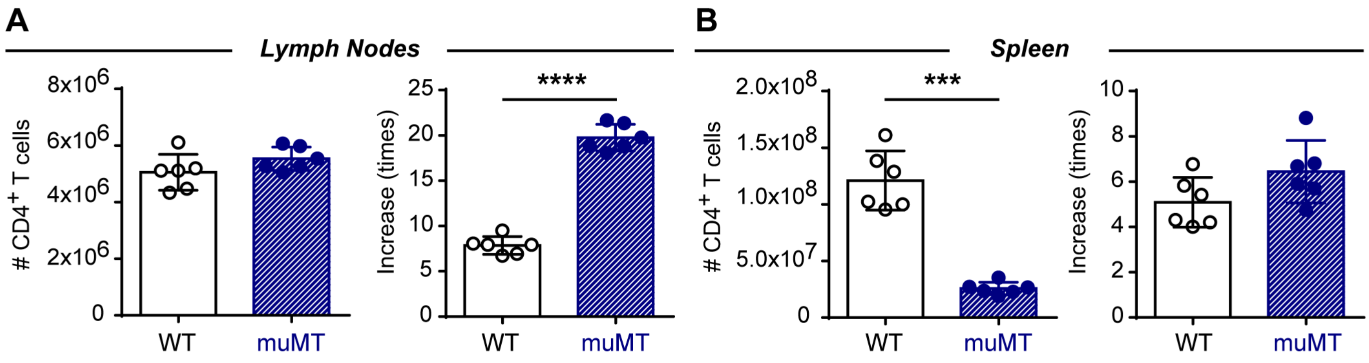

Supplementary Figure 1: Number of CD4+T cells in *T. cruzi* infected WT and muMT mice. WT (n=6, white bars) and muMT (n=6, blue bars) mice were infected with 10000 trypomastigotes of *T. cruzi* Y strain. At 15 Dpi, lymph nodes and spleen were obtained. Cells were counted in Neubauer chamber and stained with fluorochrome labelled anti-CD3 plus anti-CD4 and processed for flow cytometry. Graphics show the number of CD4+ T cells at 15 Dpi and the increase of CD4+T cell number in infected mice respect to non-infected mice in lymph nodes (A) and in the spleen (B).

1.2 Supplementary Figure 2

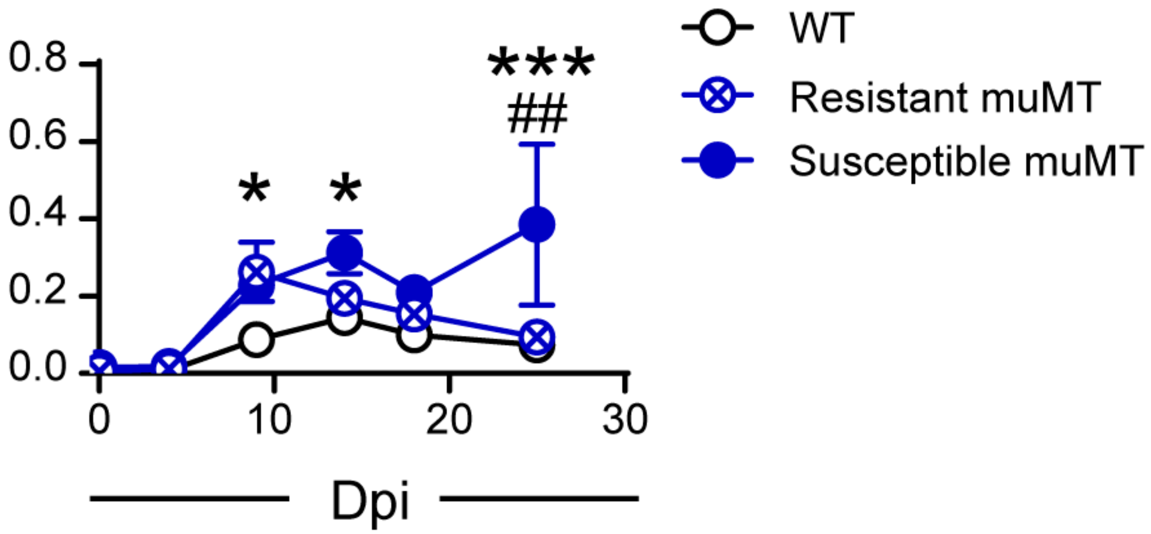

Supplementary Figure 2: Link between high plasma concentration of TNF and occurrence of death in *T. cruzi* infected muMT mice

WT (n=9, empty circles) and muMT (n=13) mice were infected with 10000 trypomastigotes of *T. cruzi* Y strain. Mice were identified and blood samples were obtained at different times post infection. By the end of the experiment (Day 40pi), each infected muMT mouse were differentiated between susceptible (n=9, blue circles) or Resistant (n=4, blue crossed circles), considering whether the mouse had died or not during the experiment. TNF was quantified in plasma samples of each infected muMT sub-group and WT mice by ELISA. The graph shows concentrations of TNF in plasma at different Dpi. Two way Anova with Bonferroni post-test * comparison between Susceptible muMT and WT, # comparison between Susceptible muMT and Resistant muMT. Experiment representative of two.

1.3 Supplementary Figure 3

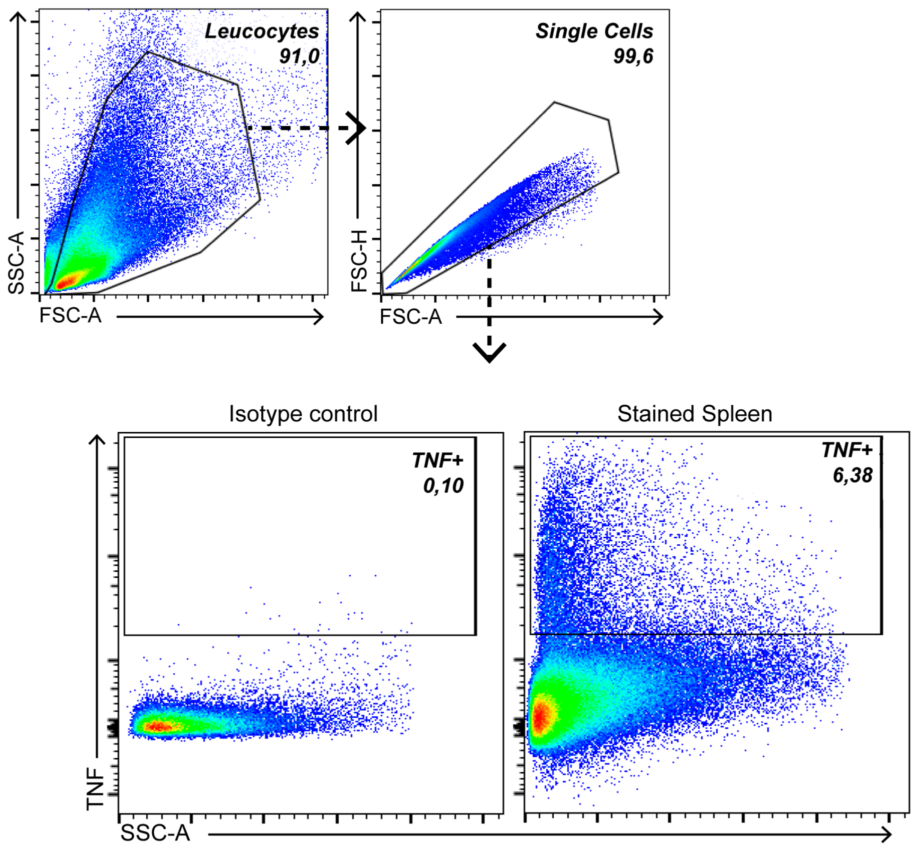

Supplementary Figure 3: Gating strategies for TNF+ cells in the spleen of *T. cruz*i infected muMT mice.

Cells from the spleen of muMT mice infected with 10.000 trypomastigotes of *T. cruzi* were obtained at 15 Dpi and processed for flow cytometry to evaluate TNF-producing cells. Dot plots of SCC-A vs FSC-A and FSC-H vs FSC-A show the gate of single leucocytes analyzed. Dot plots of TNF vs SSC-A show the gate of TNF+ single leucocytes.

1.4 Supplementary Figure 4

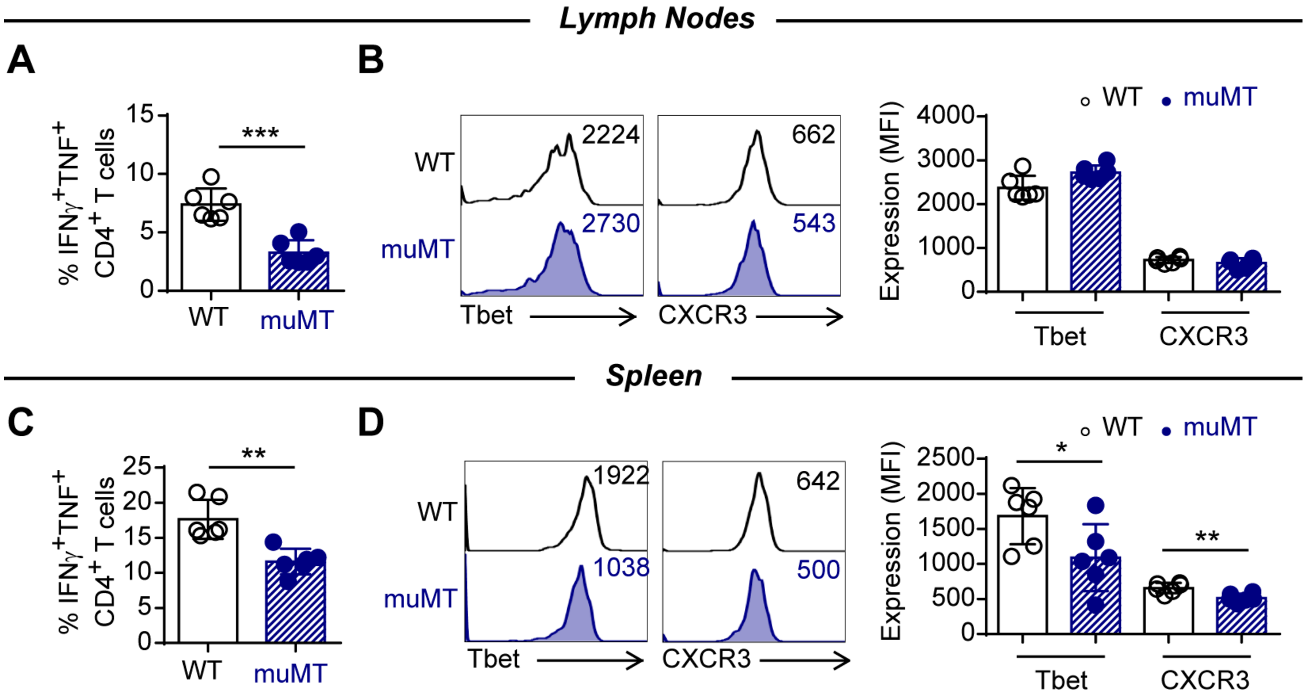

Supplementary Figure 4: Frequency of polyfunctional IFNγ⁺TNF⁺CD4+ T cells in *T. cruzi* infected WT and muMT mice.

(A, C) Percentage of $IFN\gamma^+TNF^+$ cells gated on $CD4^+CD3^+$T cells of (A) lymph nodes and (C) spleen from WT (n=6) and muMT (n=6) mice infected with 10000 trypomastigotes of *T. cruzi* obtained at 15 Dpi. Bars represent mean ± SD of the percentage $IFN\gamma^+TNF^+CD4^+CD3^+$T cells.

(B, D) Representative histograms and statistical analysis showing Tbet and CXCR3 expression on $IFN\gamma^+TNF^+CD4^+CD3^+$ T cells of (B) lymph nodes and (D) spleen from infected WT and muMT mice obtained at 15 Dpi. Bars represent mean ± SD of the molecule expression on $IFN\gamma^+TNF^+CD4^+CD3^+$, determined by geometric mean. Each symbol represents an individual mouse. (*p<0.05, **p<0.01, ***p<0.001; two-tailed t-Test).

1.5 Supplementary Figure 5

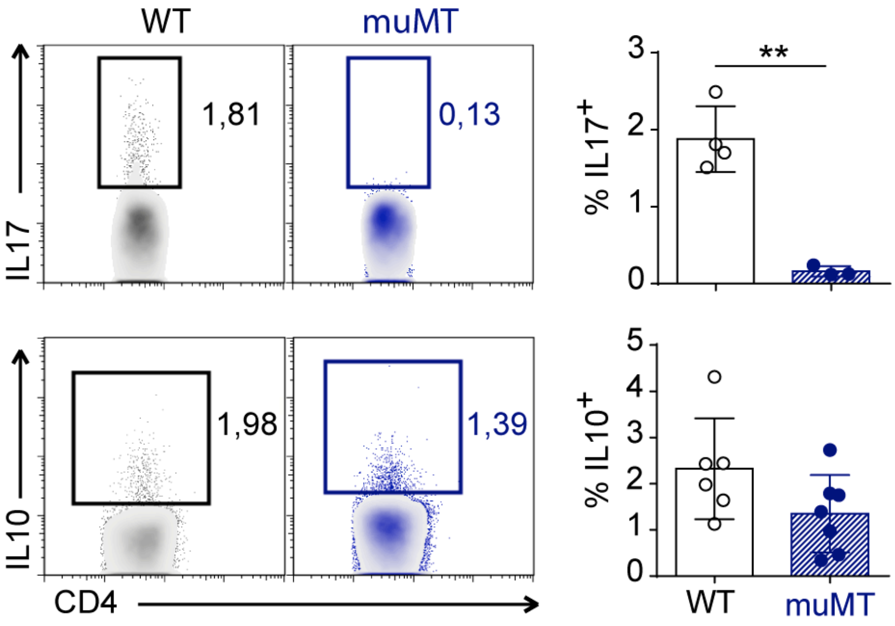

Supplementary Figure 5: Frequency of IL-17- and IL-10 producing CD4$^+$ T cells in *T. cruzi* infected WT and muMT mice.

Representative dot plot showing IL-17 and IL-10 expression in CD4$^+$CD3$^+$ T cells from the spleen of WT and muMT mice infected with 10000 trypomastigotes of *T. cruzi,* obtained at 15 Dpi; and statistical analysis. Bars represent means ± SD of the percentage of IL-17$^+$CD4$^+$CD3$^+$ T cells or IL-10$^+$CD4$^+$CD3$^+$ T cells in infected WT (IL17: n=4; IL10: n=6) and muMT (IL17: n=3; IL10: n=7) mice (**p<0.01 two-tailed t-Test).

1.6 Supplementary Figure 6

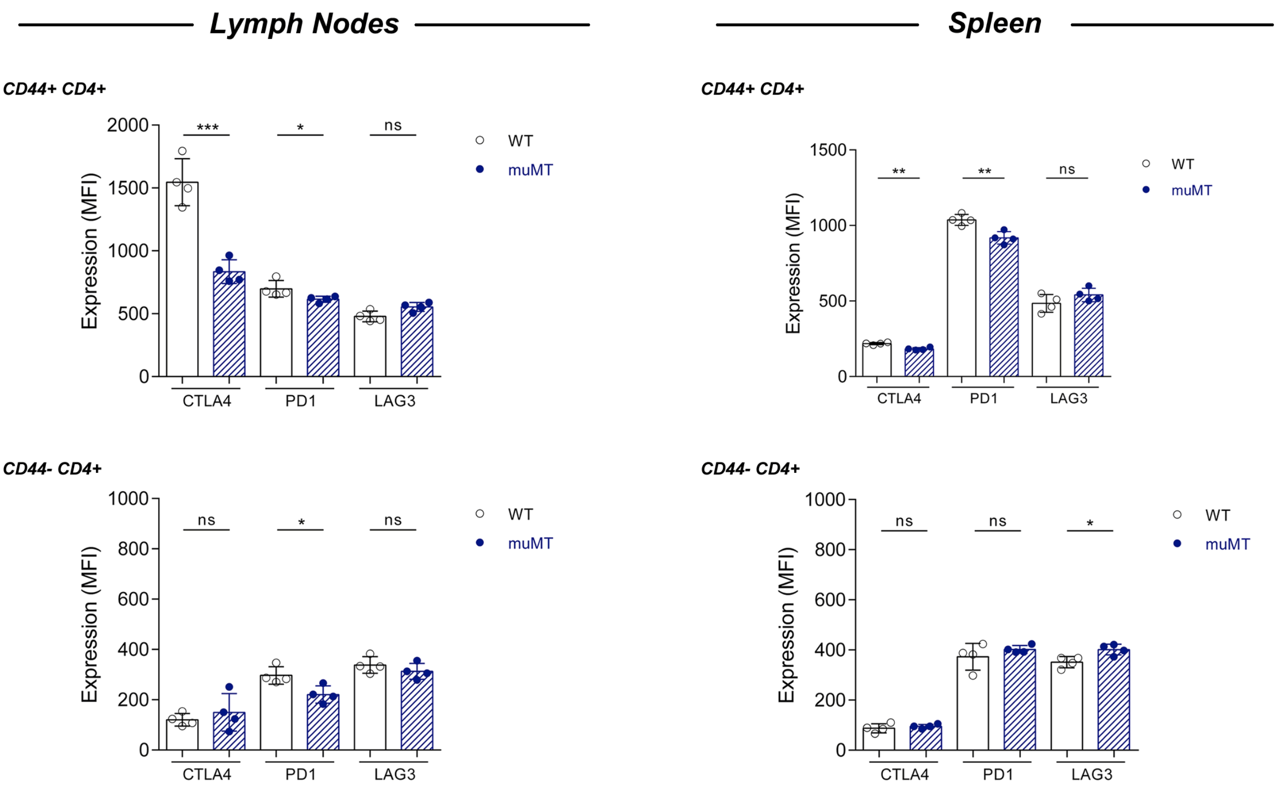

Supplementary Figure 6: Expression of inhibitory receptors on activated CD44$^+$CD4$^+$ T cells of *T. cruzi* infected WT and muMT mice

Expression (MFI) of CTLA4, PD1 and LAG3 in gated Foxp3^{neg}CD44$^+$CD4$^+$CD3$^+$T cells in lymph nodes and spleen from *T. cruzi* infected WT and muMT mice obtained at 15 Dpi. Bars represent means ± SD of the inhibitory receptor expression on Foxp3^{neg}CD44$^+$CD4$^+$CD3$^+$T cells. Each symbol represents an individual mouse. (*p<0.05, **p<0.01, ***p<0.001; ns, not significant, two-tailed t-Test). Experiment representative of two.
